# Unveiling the Mechanism of Plasma-Catalytic Low-Temperature Water–Gas Shift Reaction over Cu/γ-Al_2_O_3_ Catalysts

**DOI:** 10.1021/jacsau.4c00518

**Published:** 2024-08-13

**Authors:** Xiaoqiang Shen, Michael Craven, Jiacheng Xu, Yaolin Wang, Zhi Li, Weitao Wang, Shuiliang Yao, Zuliang Wu, Nan Jiang, Xuanbo Zhou, Kuan Sun, Xuesen Du, Xin Tu

**Affiliations:** † Key Laboratory of Low-Grade Energy Utilization Technologies and Systems, Ministry of Education, Chongqing University, Chongqing 400044, China; ‡ School of Energy and Power Engineering, Chongqing University, Chongqing 400044, China; § Department of Electrical Engineering and Electronics, University of Liverpool, Liverpool L69 3GJ, U.K.; ∥ School of Environmental and Safety Engineering, Changzhou University, Changzhou 213164, China; ⊥ School of Electrical Engineering, Dalian University of Technology, Dalian 116024, China; # Department of Electrical and Electronic Engineering, The University of Manchester, Manchester M13 9PL, U.K.

**Keywords:** plasma catalysis, nonthermal plasma, water−gas shift reaction, hydrogen production, *in situ* spectroscopy, density functional theory

## Abstract

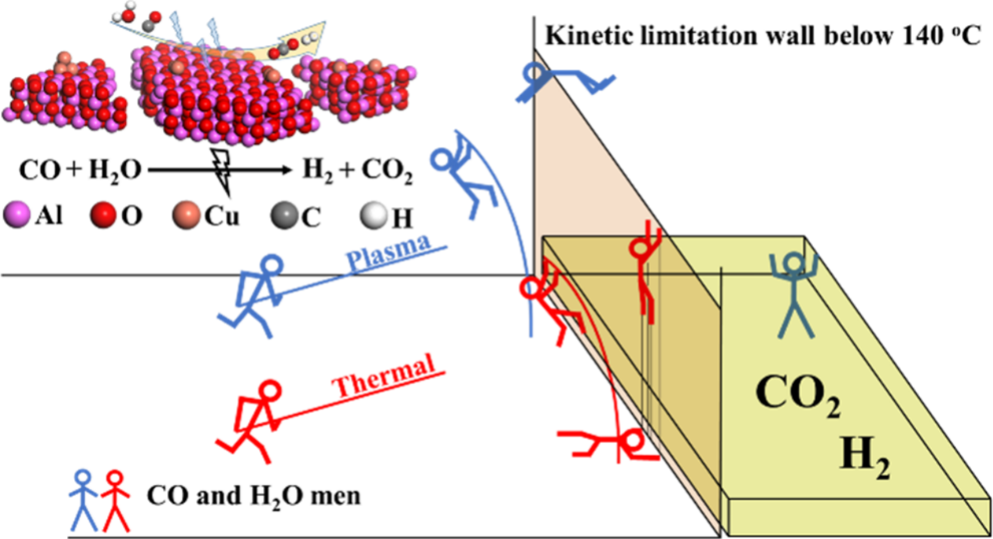

The water–gas shift (WGS) reaction is a crucial process for hydrogen production. Unfortunately, achieving high reaction rates and yields for the WGS reaction at low temperatures remains a challenge due to kinetic limitations. Here, nonthermal plasma coupled to Cu/γ-Al_2_O_3_ catalysts was employed to enable the WGS reaction at considerably lower temperatures (up to 140 °C). For comparison, thermal-catalytic WGS reactions using the same catalysts were conducted at 140–300 °C. The best performance (72.1% CO conversion and 67.4% H_2_ yield) was achieved using an 8 wt % Cu/γ-Al_2_O_3_ catalyst in plasma catalysis at ∼140 °C, with 8.74 MJ mol^–1^ energy consumption and 8.5% H_2_ fuel production efficiency. Notably, conventional thermal catalysis proved to be ineffective at such low temperatures. Density functional theory calculations, coupled with *in situ* diffuse reflectance infrared Fourier transform spectroscopy, revealed that the plasma-generated OH radicals significantly enhanced the WGS reaction by influencing both the redox and carboxyl reaction pathways.

## Introduction

The water–gas shift (WGS) reaction eq 1 is a key industrial process for generating hydrogen (H_2_). This reaction plays a crucial role in numerous industrial catalytic processes, such as ammonia synthesis, coal gasification, steam methane reforming, and hydrogen fuel cells.^1,2^ Although thermodynamically favorable at lower temperatures due to its mildly exothermic nature, the WGS reaction occurs at slower rates under these conditions, limiting CO conversion. Therefore, extensive investigations have been carried out to increase the reaction rate and yield of the WGS at low temperatures (140–300 °C).^3−6^
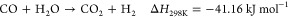
1Cu-based catalysts are commonly used in commercial low-temperature WGS reactions, often in the form of Cu/ZnO/Al_2_O_3_, where Zn serves as a copper dispersion agent to protect Cu active sites from thermal sintering and Al_2_O_3_ is used as a catalyst support to maintain stability during the reaction.^7−9^ The redox and carboxyl mechanisms, structure–activity relationships, and interactions between metals and supports in various Cu-based catalysts have been extensively studied.^10−12^ However, the kinetic limitation of the WGS reaction at low temperatures remains a challenge.

Nonthermal plasma (NTP) has great potential to overcome the kinetic limitation of the WGS reaction at lower temperatures. This emerging electrification technology, known for enhancing surface-catalyzed reactions under mild conditions,^13−17^ has attracted increasing interest for various catalytic processes, including CO_2_ hydrogenation, CO_2_ reforming of toluene, methane conversion, and ammonia synthesis.^18−23^ One of the most attractive features of NTP is its ability to generate energetic electrons and highly active species, such as OH radicals, which can activate inert molecules and break strong chemical bonds such as C=O bonds. Notably, while electrons reach high temperatures within the plasma, the bulk gas temperature remains near ambient temperature.^15,16^ This unique nonequilibrium characteristic is particularly advantageous for the WGS reaction, offering a promising route to overcome its kinetic limitations at low temperatures. Moreover, NTP can directly dissociate H_2_O molecules under ambient conditions, generating essential OH species involved in crucial WGS pathways.^24−26^ Several efforts have explored NTP-activated low-temperature WGS reactions, including increasing the electric current to increase reaction activity, applying NTP for WGS catalyst preparation, and activating WGS catalysts by NTP to promote the reaction.^27−30^ However, there have been limited investigations to date on the combination of NTP and Cu-based catalysts for catalyzing the WGS reaction at low temperatures. Additionally, the synergistic effects between plasma and the catalyst during the WGS reaction have not been fully explored. Atomistic insights into the reaction mechanism and the role of intermediates and radicals in the plasma-catalytic WGS reaction, particularly plasma-assisted surface reactions, are very limited.

Herein, plasma-enhanced catalytic WGS reactions over Cu/γ-Al_2_O_3_ catalysts with varying Cu loadings were carried out in a coaxial dielectric barrier discharge (DBD) reactor at low temperatures. These catalysts were also tested via thermal-catalytic WGS at 140–300 °C to understand the synergetic effects of plasma-catalyst coupling. Density functional theory (DFT) calculations combined with comprehensive catalyst characterization, including *in situ* plasma-coupled diffuse reflectance infrared Fourier transform spectroscopy (DRIFTS), were used to obtain new insights into the plasma-enhanced surface-catalyzed reactions in this process.

## Results and Discussion

### Catalyst Surface Physicochemical Properties

**Figure 1:**
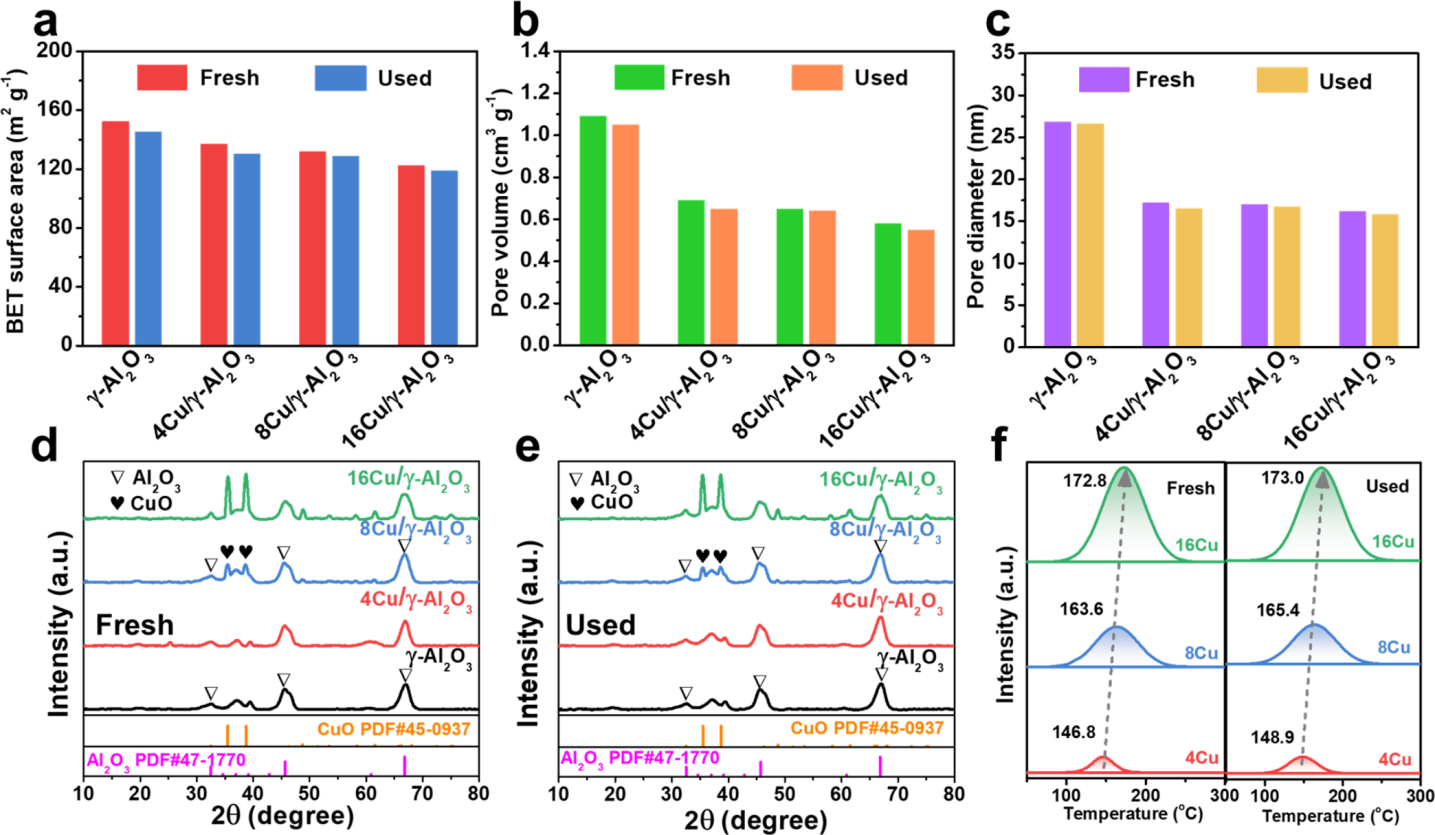
Characterization of fresh and used catalysts. (a) Specific surface areas. (b) Pore volumes. (c) Pore diameters. (d, e) XRD patterns. (f) H_2_-TPR profiles.

The specific surface area of the catalysts decreased with increasing Cu loading (Figure 1a), which could be attributed to the partial blockage of pores in γ-Al_2_O_3_ caused by Cu loading. Only very small changes were observed in the specific surface area, pore volume (Figure 1b), and pore diameter (Figure 1c) before and after the reaction, indicating the stability of the catalysts during the plasma-catalytic reaction.

The X-ray diffraction (XRD) patterns of the fresh (Figure 1d) and spent (Figure 1e) catalysts are almost identical, providing additional evidence that the catalysts remained unaffected by the reaction.^31^ High-resolution transmission electron microscopy (HRTEM) images (Figure S1a–c) show that γ-Al_2_O_3_ constitutes the majority of the catalysts, while energy-dispersive X-ray spectroscopy (EDX) analysis (Figure S1d–f) confirms the well-distributed presence of Cu nanoparticles on the surface of each catalyst.

The single peak observed in the H_2_-temperature-programmed reduction (H_2_-TPR) profiles of the Cu/γ-Al_2_O_3_ catalysts (Figure 1f) is associated with the reduction of CuO;^32^ increasing the Cu loading from 4 to 16 wt % increases the reduction temperature from 146.8 to 172.8 °C (fresh catalysts), indicating that the reducibility of the Cu/γ-Al_2_O_3_ catalyst decreases with increasing Cu loading. In addition, similar H_2_-TPR results could be found for the used Cu/γ-Al_2_O_3_ catalysts, which also illustrated that the catalysts were stable during the plasma-catalytic WGS reaction.

### Catalytic Activity Evaluation

#### Thermal- and Plasma-Catalytic WGS Reactions

**Figure 2:**
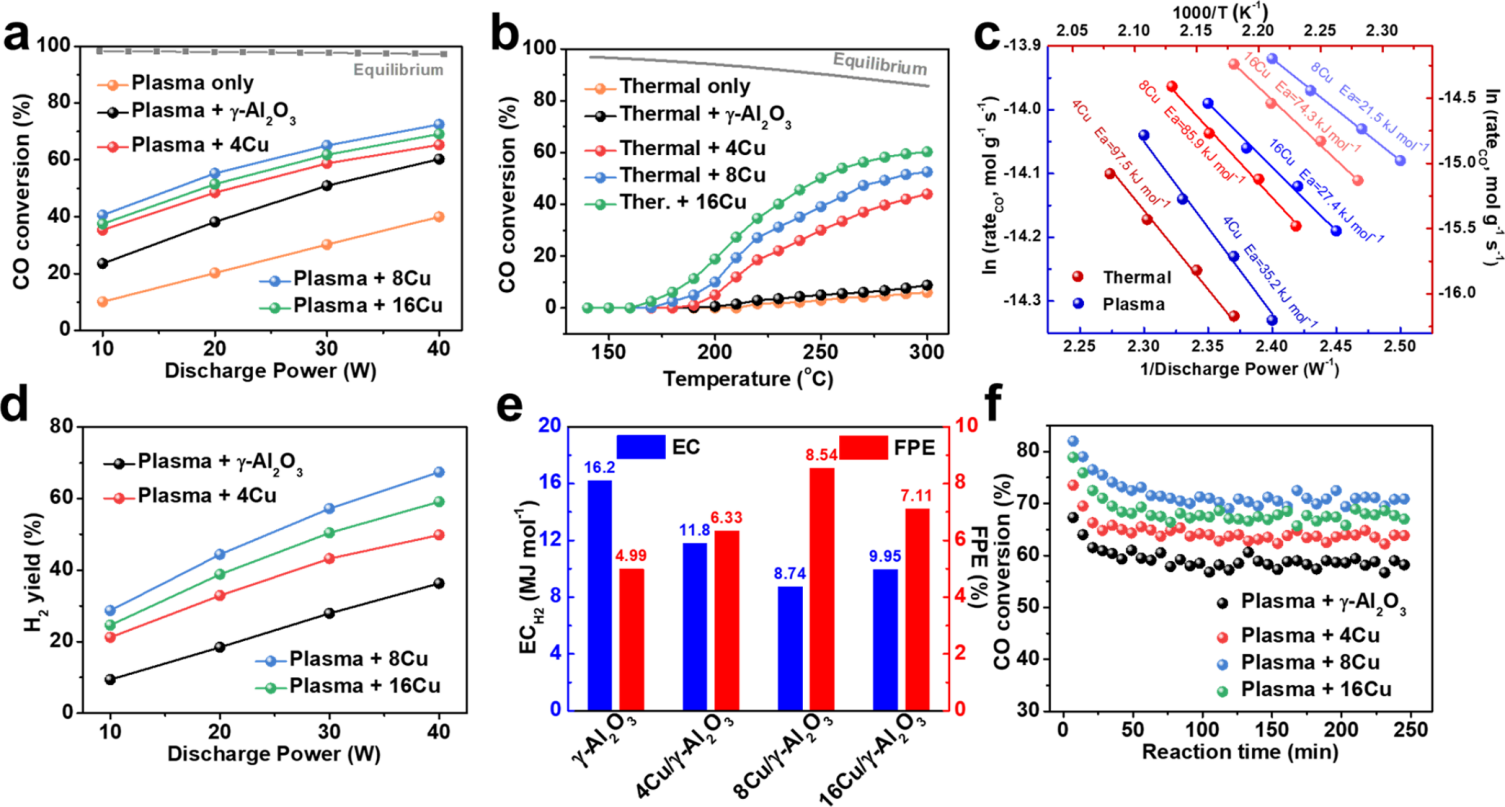
WGS reaction performance. (a) Effect of catalysts and discharge power on CO conversion in the plasma-based WGS reaction. (b) Effect of the catalyst and reaction temperature on CO conversion during the thermal-catalytic WGS reaction (reaction conditions: 10 vol % H_2_O, 10 vol % CO, and 80 vol % Ar; WHSV: 15,000 mL g_cat_^–1^ h^–1^). (c) Arrhenius plots of plasma-catalytic and thermal-catalytic processes over Cu/γ-Al_2_O_3_ catalysts (CO conversion below 15%). (d) Effect of the catalyst and discharge power on the H_2_ yield in the plasma-based WGS reaction. (e) Energy consumption and fuel production efficiency in the plasma-catalytic WGS reaction (discharge power: 40 W). (f) CO conversion as a function of time in the plasma-catalytic WGS reaction (discharge power: 40 W).

The low-temperature WGS reactions were conducted under plasma-catalytic (Figure 2a) and thermal-catalytic (Figure 2b) conditions (the experimental system is shown in Figure S2). Increasing either the discharge power in the plasma-catalytic reaction or the temperature in the thermal reaction enhanced the WGS reaction. For the thermally catalyzed reaction, the reaction did not proceed below 160 °C, as higher temperatures are required to overcome the reaction energy barrier.^29,33^ In the absence of a catalyst (thermal only), the reaction was initiated only at temperatures exceeding 210 °C. The best-performing catalyst (16Cu) improved the performance, with CO conversion detectable at temperatures as low as 170 °C. Increasing the temperature to 300 °C increased the CO conversion to 60%. However, as the temperature approached 300 °C, the CO conversion profile began to plateau, indicating that the reaction gradually became less thermodynamically favorable at these temperatures.^34^ In contrast, the use of plasma only (see Figure 2a) enabled the reaction at low temperatures (<140 °C, also shown in Figure S3) by providing energetic species to catalyze the WGS reaction, even at low discharge powers (10% CO conversion at 10 W), whereas thermal catalysis, with or without catalysts, could not enable the reaction at these temperatures. Furthermore, coupling plasma with 8Cu provided an impressive 72.1% CO conversion at ∼140 °C and 40 W. The increase in CO conversion with increasing discharge power may be attributed to the production of more energetic species in the plasma at higher discharge powers, which are crucial for the elementary reactions of the WGS mechanism cycles (e.g., −OH, H_2_O^+^, etc.).^17,35^

The important role of Cu in both plasma and thermal-catalytic processes is highlighted by the poorer performance of the γ-Al_2_O_3_-catalyzed reactions (black lines in Figure 2a,2b) compared with that of the Cu-loaded catalysts. The enhanced performance of the catalyst-coupled plasma reactions, in contrast to the reaction using plasma only, provides evidence of synergistic effects between plasma discharge and different catalysts. However, the applied voltage, current, and Lissajous figures for the different catalysts (Figure S4) are almost identical, indicating that the different Cu loadings of the catalysts packed in the DBD reactor have a negligible effect on the discharge properties. Similar findings were also reported for plasma-catalytic CO_2_ hydrogenation to methanol over CoO_*x*_/MgO catalysts with different Co loadings.^36^ In addition, the actual Cu loading and Cu dispersion of the catalyst were measured and are shown in Figure S5a,b. Due to the decay of the Cu dispersion when the Cu loading increased, the amount of surface Cu that mainly participated in the reaction process did not increase as expected (Figure S5c). This result could be responsible for the phenomenon shown in Figure 2a, where the CO conversion nearly did not change when the Cu loading increased. Furthermore, in Figure S5d–f, although the particle size increased with increasing Cu loading, the level of CO conversion did not increase. This illustrated that the Cu particle size also affects the catalytic performance of the plasma-catalytic WGS reaction. Figure S6 shows the calculated turnover frequencies (TOFs) of the plasma-catalytic and thermal-catalytic reactions. The results indicated that during plasma catalysis, both the catalyst and plasma accelerated the WGS reaction rate. Regarding thermal catalysis, although the specific surface area decreased as more Cu was loaded on the catalyst, 16Cu exhibited the highest TOF, indicating that it was more active than the other two Cu-based catalysts.^37^

Additionally, the apparent activation energies (*E*_a_) of plasma and thermal catalysis over Cu/γ-Al_2_O_3_ were calculated and are shown in Figure 2c (the *E*_a_ values for γ-Al_2_O_3_ catalysts are shown in Figure S7; the corresponding temperatures, discharge powers, and CO conversions are displayed in Tables S1–S4). The corresponding energy barriers of thermal catalysis were all approximately 60 kJ mol^–1^ higher than those of plasma catalysis, which also indicated faster reaction rates and enhancement effects of the plasma-catalytic WGS reaction at low temperatures. An obvious decrease in the reaction *E*_a_ induced by plasma has also been found for the WGS reaction over an Au-based catalyst.^38^

#### Energy Efficiency and Stability of the Plasma-Catalytic WGS Reactions

As shown in Figure 2d, the H_2_ yields of the plasma-catalytic WGS reaction were consistent with the results of CO conversion and increased with increasing discharge power. The highest H_2_ yield of 67.4% was achieved using 8Cu at a discharge power of 40 W. This reaction also had the lowest energy consumption (EC) of 8.74 MJ mol^–1^ and the highest fuel production efficiency of 8.5% for H_2_ production (Figure 2e).

During the plasma-catalytic WGS reaction, all of the catalysts exhibited stable performance over 4 h (Figure 2f). The experimental results showed that plasma catalysis outperforms thermal catalysis in catalyzing the WGS reaction at low temperatures. It has been demonstrated that plasma catalysis can accelerate the low-temperature (<140 °C) WGS reaction remarkably, whereas the same reaction is negligible in conventional thermal catalysis within the same temperature range. The combination of low-temperature and plasma-catalyst interactions can also protect the catalyst from sintering, a major issue in thermal catalysis.^39^ In addition, in a plasma reaction, the equilibrium CO conversion at low temperatures will not be limited by thermodynamic constraints, which means that in theory, complete conversion of CO could be achieved using plasma catalysis.^29^

### Reaction Mechanism

#### Investigating Strong Metal–Support Interactions (SMSIs) on the 8Cu Catalyst

**Figure 3:**
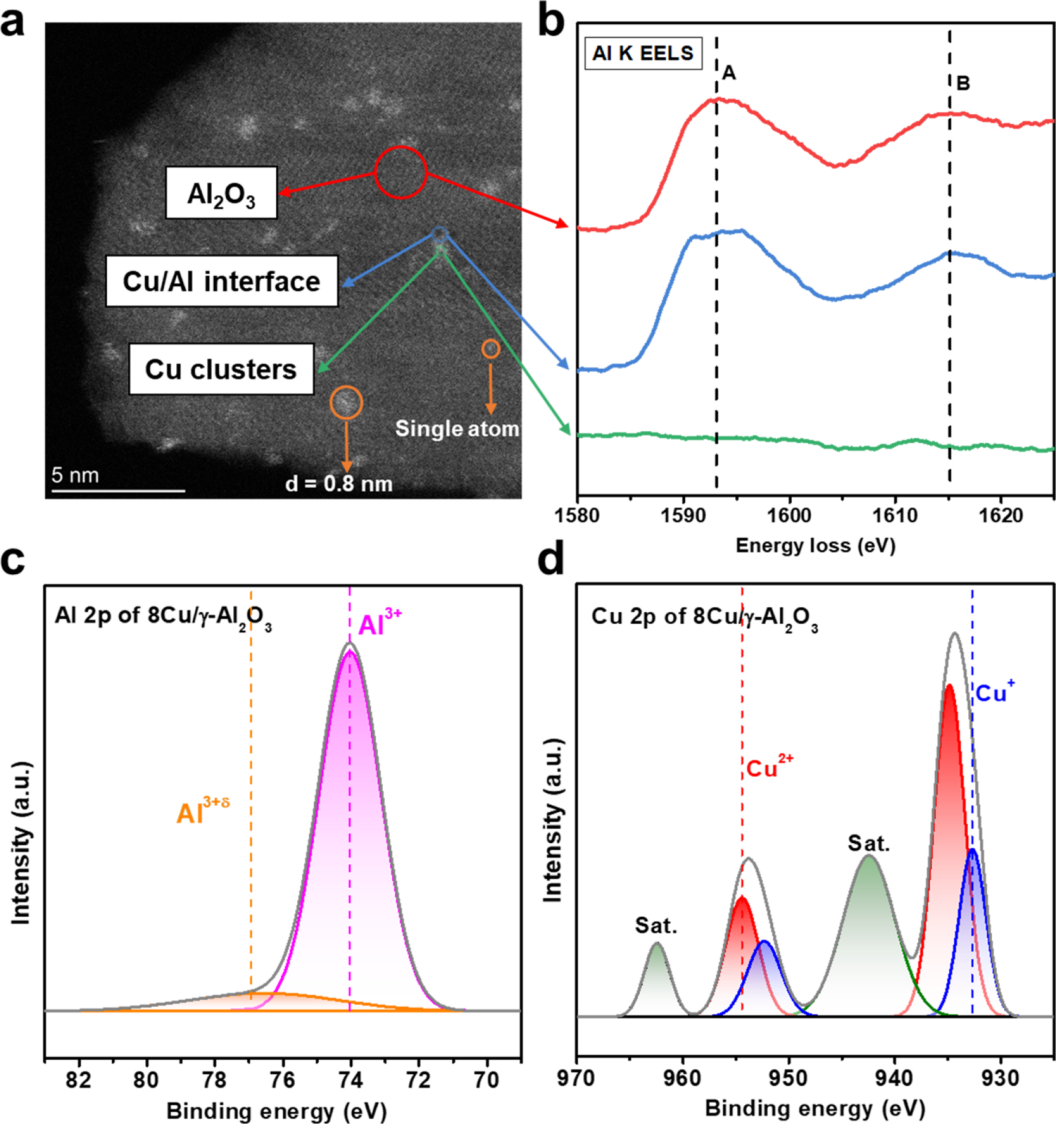
Characterization of the 8Cu catalyst after the plasma reaction. (a) HAADF-STEM image of the used 8Cu catalyst. (b) EELS spectra of Al at different surface positions. XPS spectra of (c) Al 2p and (d) Cu 2p for the 8Cu catalyst.

To understand the effect of NTP on the catalyst surface and develop a more realistic model for subsequent DFT calculations, several characterization techniques were employed to analyze the 8Cu catalyst following the plasma reaction. As shown in Figure 3a, the high-angle annular dark-field scanning transmission electron microscopy (HAADF-STEM) image clearly reveals that Cu clusters and single atoms are dispersed on the Al_2_O_3_ support. The maximum diameter of the Cu clusters is ∼0.8 nm, suggesting that they contain only a small number of Cu atoms. Based on this observation, subsequent DFT calculations will use models with small Cu clusters. Additionally, the HAADF-STEM image indicates good dispersion of Cu on the catalyst.^40,41^ Furthermore, Figure 3b presents an analysis of the Al K-edges at different surface sites using electron energy loss spectroscopy (EELS). The relative intensity ratio (A/B) of the Al K-edges slightly decreases from 2.25 for Al_2_O_3_ to 2.14 for the Cu–Al_2_O_3_ interface. This indicates that Al at the interface becomes more ionic. These findings are consistent with the X-ray photoelectron spectroscopy (XPS) results shown in Figure 3c,d, where the presence of Al^3+δ^ and Cu^0^ species suggests a strong SMSI.^42^ In summary, these results confirm the interaction between Cu clusters and the Al_2_O_3_ surface. This interaction at the interface may play a crucial role in catalyzing the WGS reaction under plasma conditions.

#### *In Situ* Plasma-Coupled DRIFTS and Optical Emission Spectroscopy (OES)

**Figure 4:**
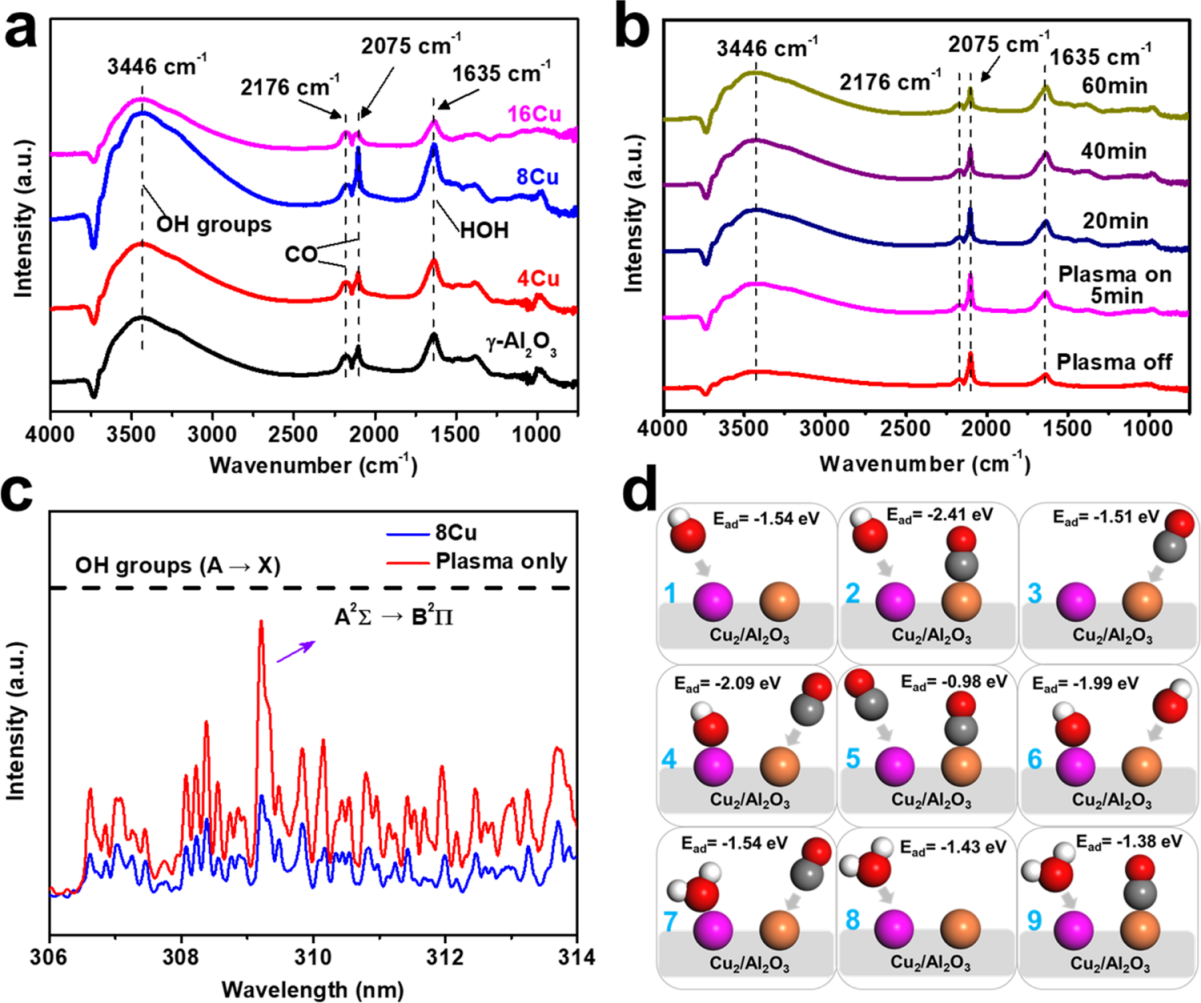
*In situ* DRIFT spectra of (a) CO and H_2_O adsorbed on the *x* Cu/γ-Al_2_O_3_ (*x* = 0, 4, 8, 16) surface at steady state and (b) CO and H_2_O adsorbed on the 8Cu/γ-Al_2_O_3_ catalyst with and without plasma discharge (total flow rate: 100 mL min^–1^ with 10 vol % H_2_O, 10 vol % CO, and 80 vol % Ar; discharge frequency and applied voltage are 500 Hz and 6.38 kV, respectively). (c) Optical emission spectra of the discharges with the 8Cu catalyst and without a catalyst (total flow rate: 100 mL min^–1^ with 10 vol % H_2_O; discharge power: 10 W). (d) Adsorption of H_2_O, CO, and OH on the Cu_2_/γ-Al_2_O_3_ catalyst surface (d1) adsorption of OH on the Al site with CO and (d2) without CO adsorbed on the Cu site; (d3) adsorption of CO on the Cu site with OH and (d4) without OH adsorbed on the Al site; (d5) adsorption of CO on the Al site with CO adsorbed on the Cu site; (d6) adsorption of OH on the Cu site with OH adsorbed on the Al site; (d7) adsorption of CO on the Cu site with H_2_O adsorbed on the Al site; (d8) adsorption of H_2_O on the Al site without CO and (d9) with CO adsorbed on the Cu site. The pink and orange balls represent the Al_3C_ and C2 sites on the Cu_2_/γ-Al_2_O_3_ catalyst surface, respectively (see Figure S9); the red, gray, and white balls represent O, C, and H atoms, respectively.

The adsorption and reaction of CO and H_2_O on the surfaces of the different catalysts were investigated through *in situ* plasma-coupled DRIFTS in the presence and absence of plasma (Figure S8). As shown in Figure 4a, sharp peaks at 1635 cm^–1^ and broad peaks centered at approximately 3446 cm^–1^ are present in the spectra of all of the catalysts and are attributed to HOH bending and OH stretching vibrations, respectively.^6,26,43,44^ Two CO peaks at 2176 and 2103 cm^–1^ are also present in all of the spectra. The peak at 2176 cm^–1^ corresponds to gaseous CO, while the 2103 cm^–1^ peak is associated with linear and bridged-bonded CO.^44−46^ The intensities of the OH peaks and the CO peak at 2103 cm^–1^ were higher when 8Cu was compared to those of other catalysts, indicating that the 8Cu catalyst surface was more enriched with adsorbed OH and CO species in the plasma environment than the other catalysts. This may help explain the results shown in Figure 2a,2d, where plasma combined with the 8Cu catalyst exhibited the best catalytic activity, as increasing the localized concentration of these species on or around the surface of the catalyst would likely facilitate and enhance the WGS reaction. In addition, Figure 4b shows the spectra of the 8Cu catalyst before and after the plasma was switched on. The intensities of the OH peaks at 1635 and 3446 cm^–1^ increased over time after switching on the plasma, suggesting that these species facilitate the WGS reaction. However, the intensity of the CO peak at 2103 cm^–1^ gradually decreased over 60 min, suggesting that adsorbed CO and OH competed for the catalyst surface sites. Over time, the surface gradually became more saturated with OH species until equilibrium was established. This explains the gradual decrease in CO conversion observed in Figure 2f within the first 60 min before reaching a steady state, as less CO adsorbs on the catalyst surface after 60 min than at the beginning of the reaction. Furthermore, the EELS results in Figure 3b suggest that the structure of Cu–O–Al at the interface may influence the transfer of charge between the catalyst surface and the reactants. This, in turn, could affect the CO conversion during the reaction.

OES was subsequently conducted to monitor the OH species in the gaseous phase during plasma activation. Figure 4c shows the typical emission spectra of H_2_O plasmas in the DBD reactor. OH (A **→** B) groups from 306 to 314 nm were detected in the plasma both with and without a catalyst; the OH (A^2^Σ **→** B^2^Π) at 309 nm produced the most intense signals in both spectra.^47,48^ The intensity of the OH signals clearly decreases in the presence of the 8Cu catalyst, which indicates that a significant portion of the OH species produced in the plasma will be adsorbed onto the catalyst surface to facilitate the WGS reaction, supporting the findings of the *in situ* plasma-coupled DRIFTS analysis.

#### Adsorption of CO, H_2_O, and OH during the WGS Reaction

DFT calculations were carried out to better understand the different mechanisms underlying the WGS reaction when using plasma catalysis versus thermal catalysis. The adsorption of CO, H_2_O, and OH on different sites on the catalyst surface was investigated first.^25,49,50^ In the plasma catalysis system, *in situ* plasma-coupled DRIFTS and OES analyses confirmed that OH species directly adsorbed onto the catalyst surface to participate in the elementary steps. The adsorption energies of H_2_O, OH, and CO on different surface sites (shown in Figure S9) are listed in Table S5. OH and H_2_O preferentially adsorb on the Al_3C_ sites, whereas CO prefers to bond with Cu atoms, particularly on the Cu sites of the Cu_1_/γ-Al_2_O_3_ and Cu_2_/γ-Al_2_O_3_ surfaces. Notably, CO exhibits a higher adsorption energy on the Cu atoms of the Al–O–Cu structure (1.45–1.52 eV on Cu_1_/γ-Al_2_O_3_ and Cu_2_/γ-Al_2_O_3_) than on the Cu tetrahedral structure of Cu_4_/γ-Al_2_O_3_ (0.80–1.27 eV). The XRD results (Figure 1d,1e) reveal that most of the Cu atoms in the 8Cu catalyst remain in the amorphous form, whereas the 16Cu catalyst contains a substantial amount of CuO crystals. Thus, the well-dispersed and amorphous CuO_*x*_ species in the 8Cu catalyst can generate more Al–O–Cu structures that are beneficial for CO adsorption, which is consistent with the *in situ* plasma-coupled DRIFTS analysis (Figure 4a,4b), which shows the highest adsorption peak on the 8Cu catalyst surfaces. Subsequently, the coadsorption of CO and OH on the Cu_2_/γ-Al_2_O_3_ surface (Figure 4d) was evaluated. Figure 4d1,d2 shows that when CO is adsorbed on a Cu site, the adsorption of OH on the adjacent Al_3C_ site is enhanced. Similarly, the adsorption of CO on a Cu site is enhanced when OH is already adsorbed on an adjacent Al_3C_ site (Figure 4d3,d4). These findings suggest a synergistic effect between the adsorption of CO and OH, consistent with the results in Figure 4d1, where the adsorption of CO and OH on the 8Cu catalyst is more pronounced than that on the other catalysts. Furthermore, when the Cu site is occupied by CO, the adjacent Al site more readily binds with OH than with CO (Figure 4d2,d5). As shown in Figure 4d4,d6, the adsorption energies of OH and CO on Cu are comparable when an adjacent Al has already adsorbed OH. This finding implies that the adsorption of OH and CO on Cu sites becomes competitive when OH is abundant on the catalyst surface. This theoretical observation is consistent with the *in situ* plasma-coupled DRIFTS characterization results (Figure 4b), which reveal a slight decrease in the adsorption intensity of the CO and an increase in the OH peak upon plasma activation.

We also examined the coadsorption of CO and H_2_O on the catalyst for the WGS reaction using thermal catalysis (Figure 4d1–d9). H_2_O was used in these calculations, as OH is unlikely to form in the gas phase at the temperatures used in these reactions. The results in Table S5 show that H_2_O is more favorably adsorbed on the Al_3C_ sites of the Cu_*x*_ surface than on the other sites. The coadsorption results are shown in Figure 4d4,d7–d9. The adsorption energies of H_2_O and CO hardly change when CO and H_2_O are preadsorbed on the catalyst surface. No promotional or competitive influence was found during the coadsorption of CO or H_2_O.

#### WGS Reaction Pathways Using Plasma Catalysis and Thermal Catalysis

**Figure 5:**
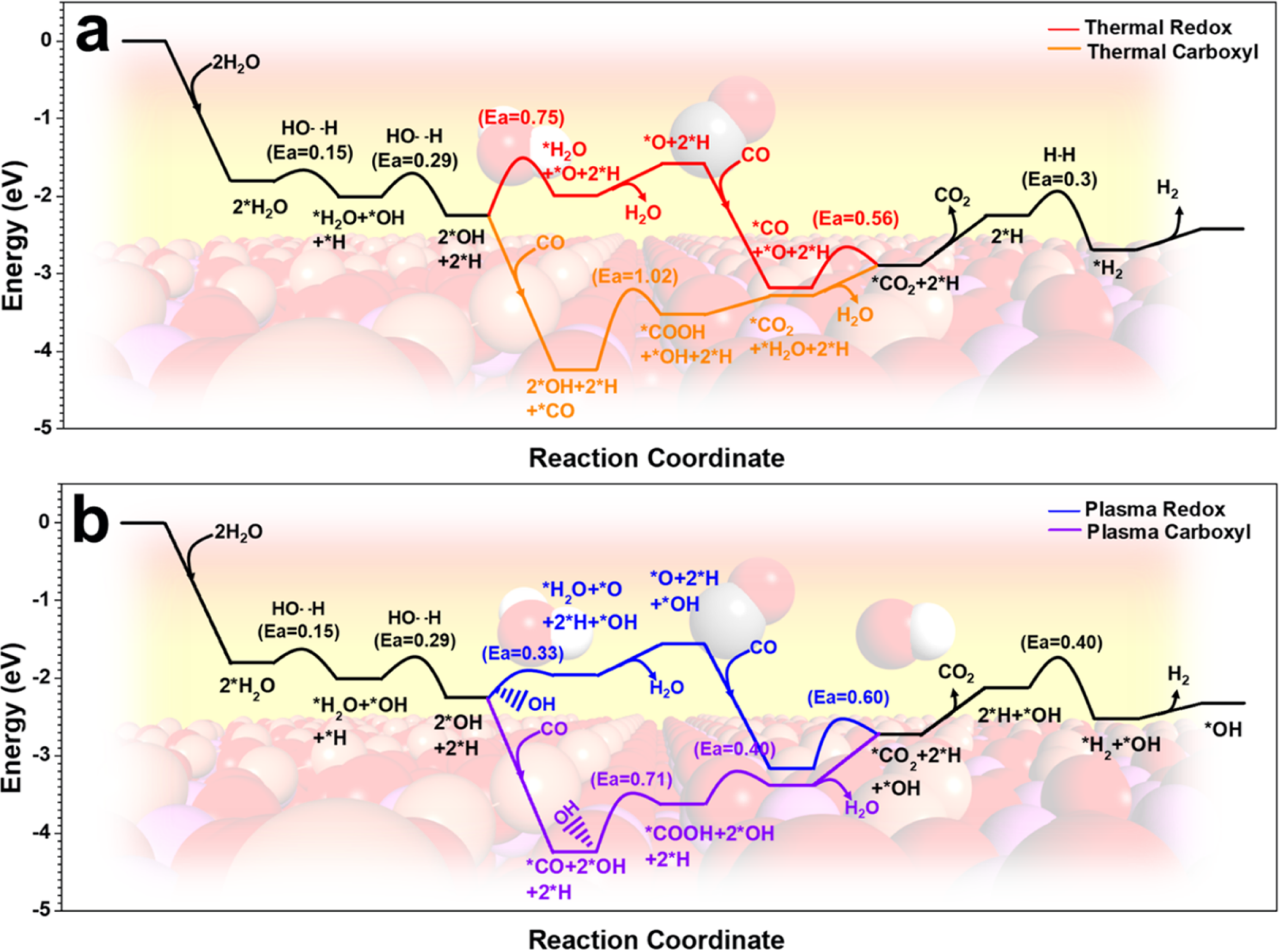
(a) Thermal and (b) plasma reaction routes and energy profiles of the WGS reaction on the Cu_2_/γ-Al_2_O_3_ catalyst (*X represents the adsorbed species). Atom color code: O, red; H, white; Cu, orange; Al, pink; C, gray.

In this section, we investigated the WGS reaction mechanisms for both thermal and plasma-catalytic processes using DFT calculations following two well-reported mechanisms: the redox mechanism and the carboxyl mechanism (Table S6). The reaction routes over the Cu_2_/γ-Al_2_O_3_ catalyst are shown below (Figure 5) to demonstrate the differences between the thermal and plasma-catalytic processes (the energy profiles of the Cu_1_/γ-Al_2_O_3_ and Cu_4_/γ-Al_2_O_3_ catalysts are listed in Tables S7 and S8). During the thermal process (Figure 5a), two H_2_O molecules were adsorbed on the surface and dissociated. Notably, the energy barrier of water decomposition (0.29 eV) to *OH and *H on the Cu_2_/γ-Al_2_O_3_ surface is lower than that on a pure Cu surface (1.36 eV), which is possibly attributed to the promotional effect of the metal–metal oxide support interaction.^10^ Along the thermal-redox route, the two *OH groups react with each other (with an energy barrier of 0.75 eV) to generate *O and *H_2_O, which were found to be the rate-determining steps (RDS) for the redox route. Afterward, the produced *O reacts with the adsorbed *CO to generate *CO_2_, overcoming an energy barrier of 0.56 eV. Along the thermal-carboxyl mechanism, one of the *OH species reacts with *CO to produce *COOH, overcoming an energy barrier of 1.02 eV. Afterward, the *COOH reacted with the other *OH to produce *CO_2_ and *H_2_O. Following the desorption of *CO_2_, the remaining two *H species combined with each other to generate *H_2_. Eventually, H_2_ molecules are released from the surface.

In the plasma-catalytic WGS reaction system, two main promotional effects were considered, namely, the vibrational activation of gaseous reactants and the generation of reactive radicals.^51,52^ However, as shown in Figure 5a, the reactants, including H_2_O and CO, are both easily adsorbed on the surface. The rate-determining steps for the thermal-redox and carboxyl routes involve interactions between two surface adsorbed species. Thus, the vibrational activation effect of gaseous reactants is negligible in the plasma-catalytic WGS reaction.

Therefore, reactive radicals are believed to participate in the reaction and accelerate the key steps. In Figure 5b, we introduce OH radicals, which were found in the OES spectra, into the reactions. First, two H_2_O molecules were adsorbed on the surface and dissociated. Along the plasma-redox route, one of the surface *OH species reacts with an OH radical (0.33 eV energy barrier) from the gas phase to produce *O and *H_2_O. Later, the *O species reacted with the adsorbed *CO to generate *CO_2_, with a 0.60 eV energy barrier. Moreover, during the plasma-carboxyl cycle, adsorbed *CO reacts with OH to produce *COOH, which is accompanied by a 0.71 eV energy barrier. Afterward, *COOH reacted with one of the surface *OH species to generate *CO_2_ and *H_2_O. Following the desorption of *CO_2_, the remaining two *H species combined with each other to generate *H_2_ and desorbed. At the end of the plasma-enhanced path, an *OH species remained on the surface and further participated in the reactions (shown in Figure S10). The energy profiles clearly show that the rate-determining steps of the WGS reaction are significantly accelerated by the participation of OH radicals. The activation energies required for the generation of *O (the RDS of the redox path) and *COOH (the RDS of the carboxyl path) decreased by 0.42 eV (0.75 to 0.33 eV) and 0.31 eV (1.02 to 0.71 eV), respectively.

## Conclusions

Comprehensive experimental and theoretical investigations of plasma-catalytic and thermal-catalytic WGS reactions in this work clearly illustrate that incorporating plasma technology into low-temperature WGS reactions considerably improves the performance over thermal catalysis alone. We found that the best-performing catalysts for the two catalytic processes (plasma catalysis vs thermal catalysis) were different for different Cu loadings. The highest CO conversion (72.1%) and hydrogen yield (67.4%) were achieved when 8Cu was coupled to the plasma at ∼140 °C, while the same reaction was negligible in conventional thermal catalysis at the same temperature. These experimental results demonstrate that plasma catalysis is superior to thermal catalysis for catalyzing the WGS reaction at low temperatures. The DFT results, coupled with *in situ* plasma-coupled DRIFTS and comprehensive catalyst characterization, show that the 8Cu catalyst contains more amorphous Al–O–Cu surface structures than the other catalysts, which is beneficial for the WGS reaction. The OH radicals produced in plasma enhance the WGS reaction by altering both the redox and carboxyl pathways. These results represent a successful attempt to combine plasma and Cu catalysts to catalyze the WGS reaction at low temperatures and provide new and valuable insights into the reaction mechanisms in the plasma-catalytic WGS reaction using DFT modeling coupled with *in situ* plasma-coupled DRIFTS and OES.

## Experimental Section

### Experimental Setup

In this study, a typical cylindrical DBD plasma reactor was used for plasma-catalytic WGS, as shown in Figure S2b. The discharge gap was 2.5 mm, with a discharge length of 90 mm. The inner electrode was a stainless rod connected to a high-voltage output, and the outer electrode was an Al foil grounded via an external capacitor *C*_ext_ (0.47 μF). The DBD reactor was connected to a high-voltage power supply (CTP-2000K) with a maximum applied voltage of 30 kV and an adjustable frequency of 5 to 20 kHz. The frequency of the power supply was fixed at 10 kHz in this study. The current and applied voltage signals were measured by using a current transformer (P6039A, Pintech, China) and a high-voltage probe (PT320, Pintech, China), respectively. All of the electrical signals were recorded using a digital oscilloscope (TDS-2014C). The temperatures were measured by a thermocouple thermometer, with the probe touching the outer surface of the quartz glass tube. A detailed description of the experimental setup can be found in the second part of the Supporting Information.

### Catalyst Synthesis

The *x* wt % Cu/γ-Al_2_O_3_ (*x* = 4, 8, and 16) catalysts were prepared using the incipient wetness impregnation method with Cu(NO_3_)_2_·3H_2_O as a metal precursor. Cu(NO_3_)_2_·3H_2_O was initially dissolved in 7 mL of deionized water and subsequently added to 5 g of γ-Al_2_O_3_ powder. The resulting slurry was stirred for 1 h, followed by 30 min of oscillation using an ultrasonic oscillator and overnight impregnation. Subsequently, the slurry was dried at 105 °C for 12 h, followed by calcination at 550 °C for 5 h. Finally, the obtained catalyst was crushed and sieved into 40–60 mesh. The catalysts were denoted as 4Cu, 8Cu, and 16Cu in reference to their respective wt % Cu loading.

### Catalyst Characterization

Brunner–Emmett–Teller (BET) measurements via N_2_ adsorption–desorption isotherms were conducted to determine the pore size and specific surface area of the catalysts using a surface area analyzer (Micromeritics ASAP 2460). XRD patterns of the catalysts were recorded by an X-ray diffractometer (Bruker, D8 ADVANCE) equipped with Cu Kα radiation (40 kV tube voltage and 40 mA tube current) in the 2θ range between 10 and 80°. HRTEM was performed on a JEM 2100F high-resolution transmission electron microscope. Energy-dispersive X-ray spectroscopy (EDX) was performed on a Talos F200X energy-dispersive spectrometer with an accelerating voltage of 200 kV. The reducibility of the catalysts was evaluated using H_2_-TPR on a fully automated chemisorption analyzer (Finesorb3010). Before each run, 0.2 g of the catalyst was added to the fixed bed and preheated to 200 °C under an N_2_ flow (50 mL min^–1^) for 1 h to remove physisorbed and/or weakly bound species. After cooling to room temperature, the catalyst was heated from room temperature to 800 °C at a heating rate of 10 °C min^–1^ under a 5 vol % H_2_/N_2_ gas mixture (total flow rate: 50 mL min^–1^). HAADF-STEM analysis was performed by using a JEM 2100F high-resolution transmission electron microscope. The EELS analysis was conducted on an FEI Titan G2 60–300 microscope equipped with a Gatan Imaging Filter system operated at 200 kV. XPS profiles were recorded using an ESCALAB 250Xi photoelectron spectrometer (Thermo Fisher Scientific) with Al Kα radiation (*h*ν = 1486.6 eV).

*In situ* plasma-coupled DRIFTS was used to investigate the adsorption of H_2_O and CO on the Cu/γ-Al_2_O_3_ catalysts by using a Nicolet 50 Fourier transform infrared (FTIR) spectrometer (Figure S8). The spectra were collected with and without plasma discharge to examine the effect of plasma on the reaction. Before adsorption, the catalyst was pretreated for 1 h in Ar at 400 °C. Then, the catalyst was cooled to room temperature and stabilized for 10 min, after which background spectra were collected. A mixture of CO (10 vol %), H_2_O (10 vol %), and Ar was injected into the reaction cell, and the spectra were collected. Details of the *in situ* plasma-coupled DRIFTS are available in the fourth part of the Supporting Information.

OES diagnostics were performed to measure the emission spectra of the discharge in a gas flow containing 10 vol % H_2_O. The total gas flow rate was 100 mL min^–1^. The spectrometer (USB2000+, Ocean Optics) has a wavelength range from 180 to 1100 nm and a spectral resolution of 1.4 nm at the full width at half-maximum. The fiber was positioned close to the center of the quartz reactor axis and at a distance to maximize the light emission collected from the DBD reactor.

## Supporting Information Available

Details of the *in situ* plasma-coupled DRITFS experiments; HRTEM and EDX results; electrical signals from the DBD experiments; calculations of turnover frequency; calculation equations of catalytic performance, and DFT-derived theoretical results (PDF)
