# Supplementary material for: Unveiling the Mechanism of Plasma-Catalytic Low-Temperature Water–Gas Shift Reaction over Cu/γ-Al2O3 Catalysts
Source: JACS Au. 2024 Aug 13;4(8):3228–37. doi: 10.1021/jacsau.4c00518 (PMC11350726; doi:10.1021/jacsau.4c00518)
Supplement: Supplementary file 1 — au4c00518_si_001.pdf [file au4c00518_si_001.pdf]

## Supporting Information

# Unveiling the Mechanism of Plasma-Catalytic Low-Temperature Water-Gas Shift Reaction over Cu/γ-$Al_2O_3$ Catalysts

Xiaoqiang Shen[a, b], Michael Craven[c], Jiacheng Xu[d], Yaolin Wang[c], Zhi Li[a, b], Weitao Wang[c], Shuiliang Yao[d], Zuliang Wu[d], Nan Jiang[e], Xuanbo Zhou[f], Kuan Sun[a, b], Xuesen Du[a, b, *], Xin Tu[c, *]

[a] Key Laboratory of Low-grade Energy Utilization Technologies and Systems, Ministry of Education, Chongqing University, Chongqing 400044, China

[b] School of Energy and Power Engineering, Chongqing University, Chongqing 400044, China

[c] Department of Electrical Engineering and Electronics, University of Liverpool, Liverpool L69 3GJ, UK

[d] School of Environmental and Safety Engineering, Changzhou University, Changzhou 213164, China.

[e] School of Electrical Engineering, Dalian University of Technology, Dalian, 116024, China

[f] Department of Electrical and Electronic Engineering, The University of Manchester, Manchester M13 9PL, UK

* **Corresponding authors**

xuesendu@cqu.edu.cn (Xuesen Du)

xin.tu@liverpool.ac.uk (Xin Tu)

**Table of Contents**


1. **Catalyst characterization**
2. **Additional experimental details and methods**
    - 2.1. Experimental details
    - 2.2. Catalyst volume fraction
    - 2.3. Temperatures of plasma-catalysis
    - 2.4. Electrical results
3. **Catalytic tests of catalysts**
    - 3.1. Calculation methods of catalytic performance
    - 3.2. Calculation methods of kinetics
    - 3.3. Calculation methods of energy consumption
4. ***In situ* DRIFTS experiments**
5. **DFT calculations**
    - 5.1. Calculation details
    - 5.2. Calculation results

## 1. Catalyst characterization

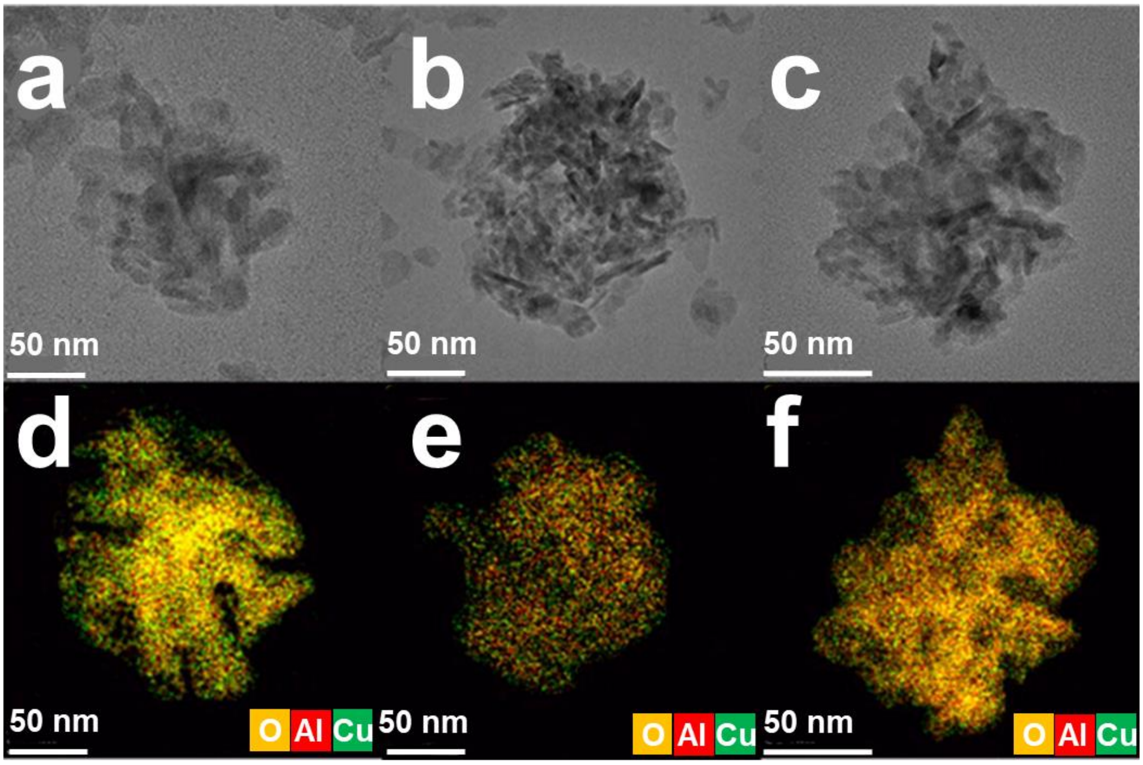


**Figure S1.** HRTEM images (a-c) and EDX pictures (d-f) of 4, 8, 16Cu fresh catalysts.

## 2. Additional experimental details and methods

### 2.1. Experimental details

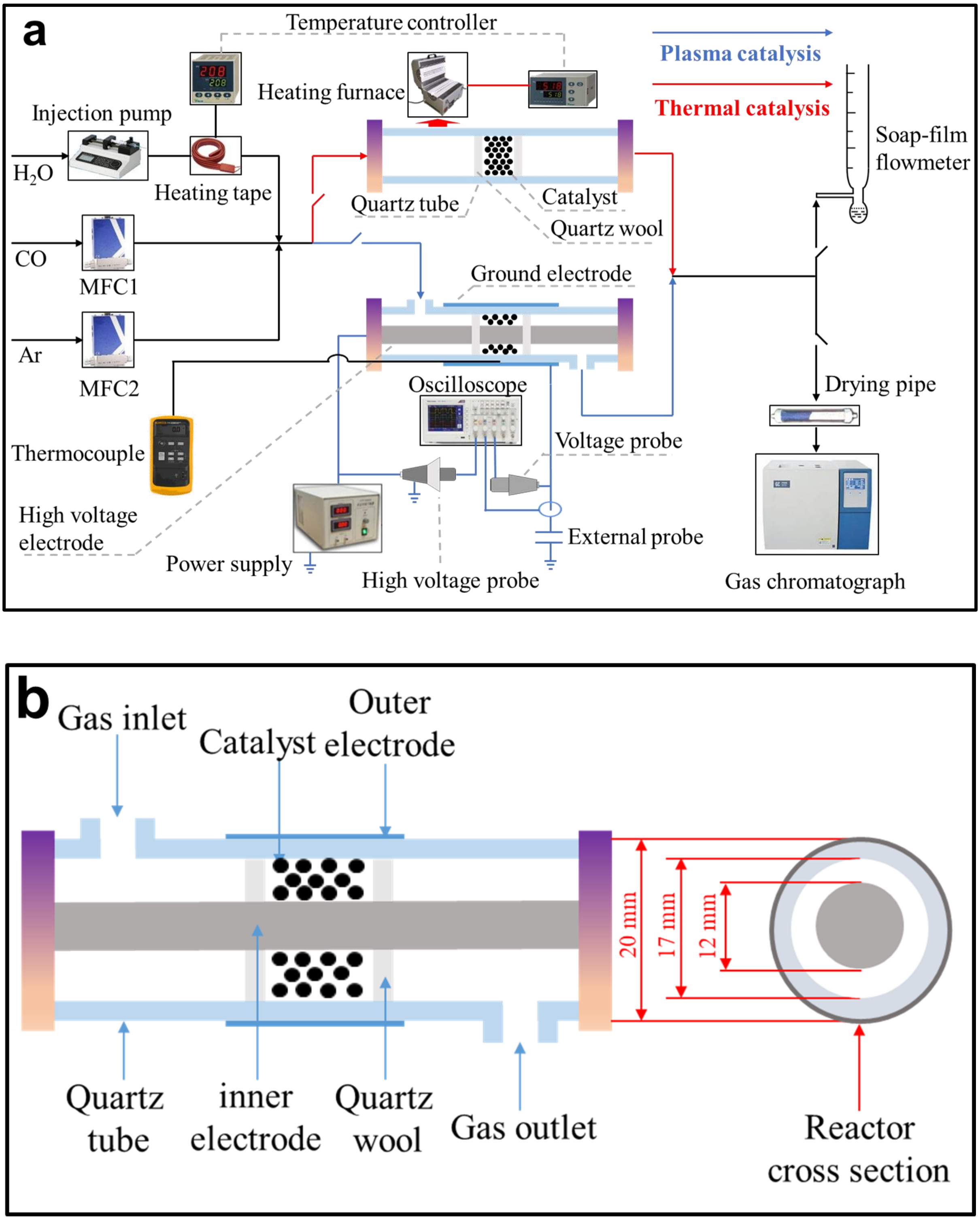


**Figure S2.** (a) Schematic of thermal-catalytic and plasma-catalytic experimental setup. (b) Schematic of and DBD reactor.

For WGS reaction test. a total of 0.4 g Cu/γ-$Al_2O_3$ catalyst (40-60 mesh) was partially packed (8%, calculation details can be found in 2.2 of supporting information) in the discharge gap. A mixed flow of CO, argon, and water vapor with a total flow rate of 100 mL/min (10 vol.% $H_2O$, 10 vol.% CO and 80 vol.% Ar) was used in this study. While the weight hourly space velocity (WHSV) was set as 15000 mL $g_{cat}^{-1}$ $h^{-1}$. The water vapor (140 $^oC$) was generated by passing deionized water successively through an injector, injection pump and a length of tube heated by heating tapes. After the mixure with CO and Ar, the feed gas entered the thermal or plasma reactor to conduct the WGS reaction. Later, the reaction products were passed through a drying pipe to remove any unreacted water vapor. Finally, the products were analyzed by a gas chromatograph (Techcomp 7900) equipped with two thermal conductivity detectors (TCDs). Besides, the soap-film flowmeter was used to measure the post-reaction product volume to ensure the accuracy of CO conversion calculation. While the thermocouple was used to acquire the temperature of plasma-catalysis during the reaction. Prior to each experiment, the Cu/γ-$Al_2O_3$ catalysts were reduced under an $N_2$-$H_2$ flow (10 vol.% $H_2$) with a total flow rate of 50 mL/min at 300 $^oC$ for 3 h. For comparison, thermal catalytic WGS reactions were carried out in a fixed-bed reactor that had the same dimensions as the quartz tube used in the DBD reactor. All experimental data were sampled and measured at least three times to ensure accuracy and reliability.

## 2.2. Catalyst volume fraction

The catalyst volume fraction ($\alpha$) in the packed discharge area can be calculated by:[1]

$$\alpha = \frac{V_{catalyst}(\mathrm{cm}^3)}{V_{discharge}(\mathrm{cm}^3)} \tag{1}$$

Where $V_{discharge}$ is total volume of the discharge area (1.14 $cm^3$ in this work), while $V_{catalyst}$ was measured by adding 0.4 g catalyst ($Al_2O_3$ or Cu/$Al_2O_3$) — the amount of catalyst used to pack the discharge area for the WGS reactions — to a measuring cylinder and then slowly adding

deionized water with a calibrated, adjustable volume pipette. This was done until the catalyst was completely submerged and the water level reached the same volume as the discharge area of the reactor. Each addition of water was measured using readings from the pipette. The total volume of water added was used to determine $V_{catalyst}$. In this work, the measured $\alpha$ for $Al_2O_3$, 4Cu/γ-$Al_2O_3$, 8Cu/γ-$Al_2O_3$ and 16Cu/γ-$Al_2O_3$ catalysts were 0.12, 0.08, 0.08 and 0.08, respectively. In addition, the length of the catalyst bed and overall length of the reactor are 0.25 cm and 21.3 cm, respectively.

## 2.3. Temperatures of plasma-catalysis

The temperatures of plasma-catalytic WGS reactions were measured by the thermocouple (displayed in Figure S2a). The results in Figure S3 indicated that the temperatures of plasma-catalysis were below 140 $^{o}$C during the reaction process.

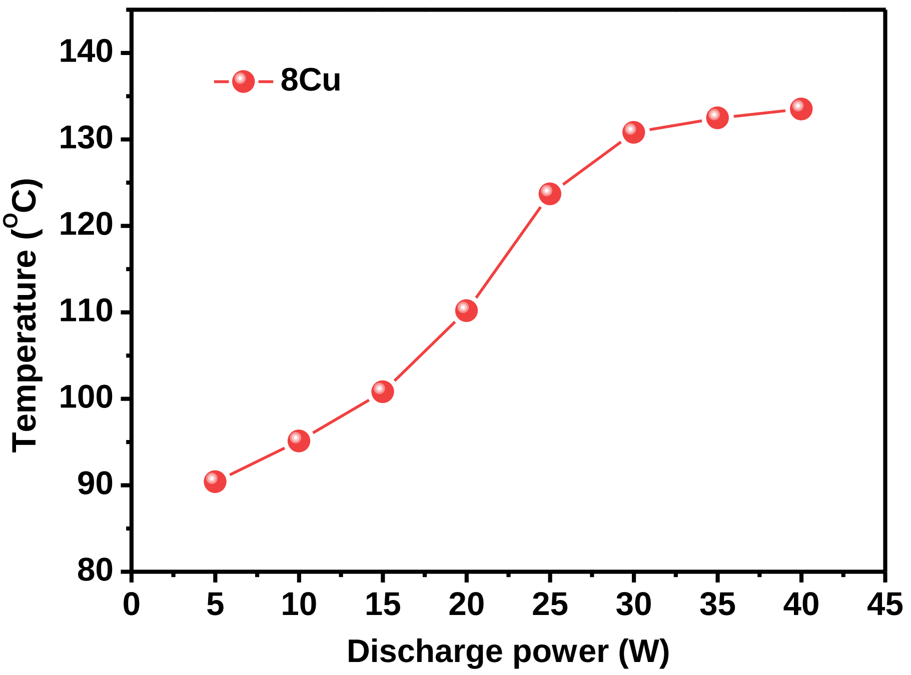


**Figure S3.** Reaction temperatures as a function of discharge power in plasma-catalytic reaction.

## 2.4. Electrical results

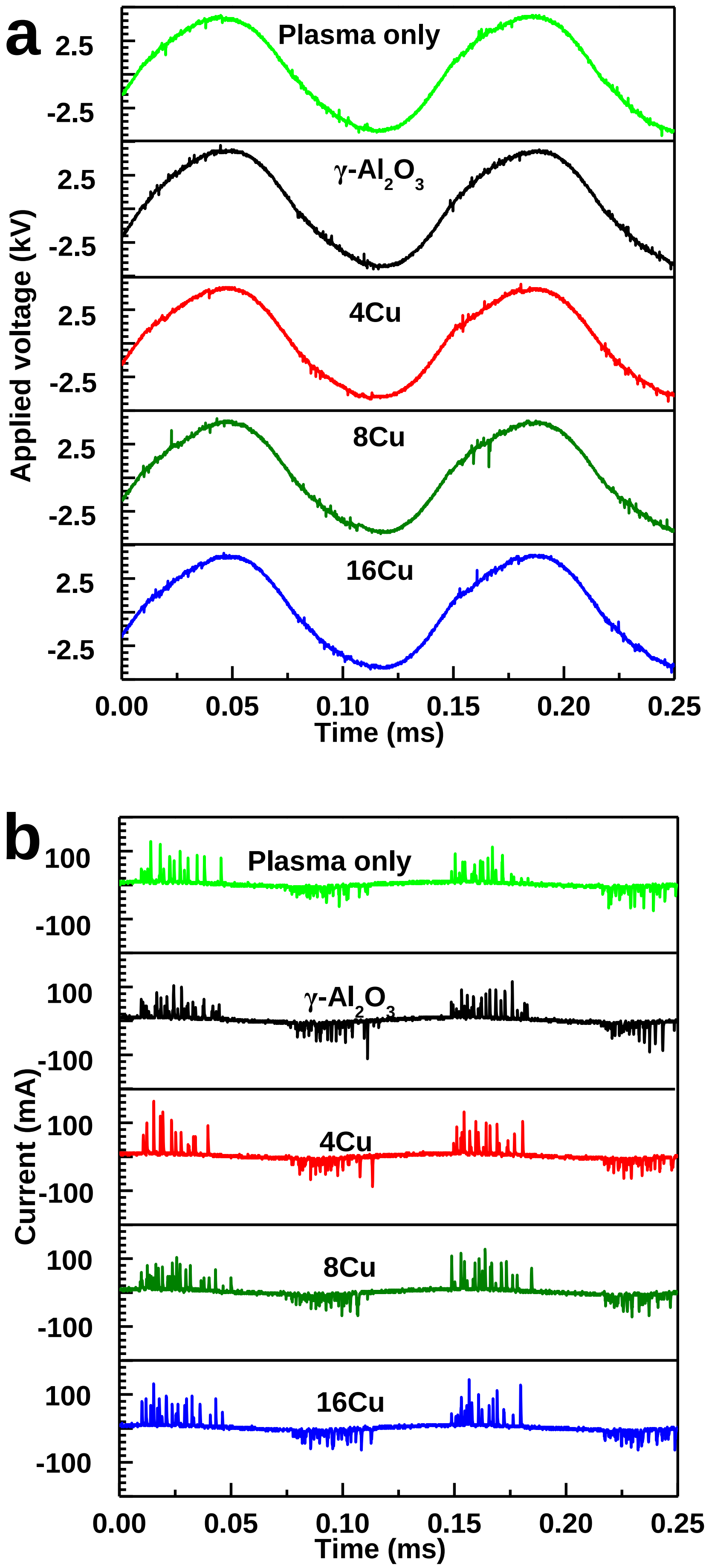
a
Plasma only
γ-Al2O3
4Cu
8Cu
16Cu
Applied voltage (kV)
2.5
-2.5
0.00
0.05
0.10
0.15
0.20
0.25
Time (ms)
b
Plasma only
γ-Al2O3
4Cu
8Cu
16Cu
Current (mA)
100
-100
0.00
0.05
0.10
0.15
0.20
0.25
Time (ms)

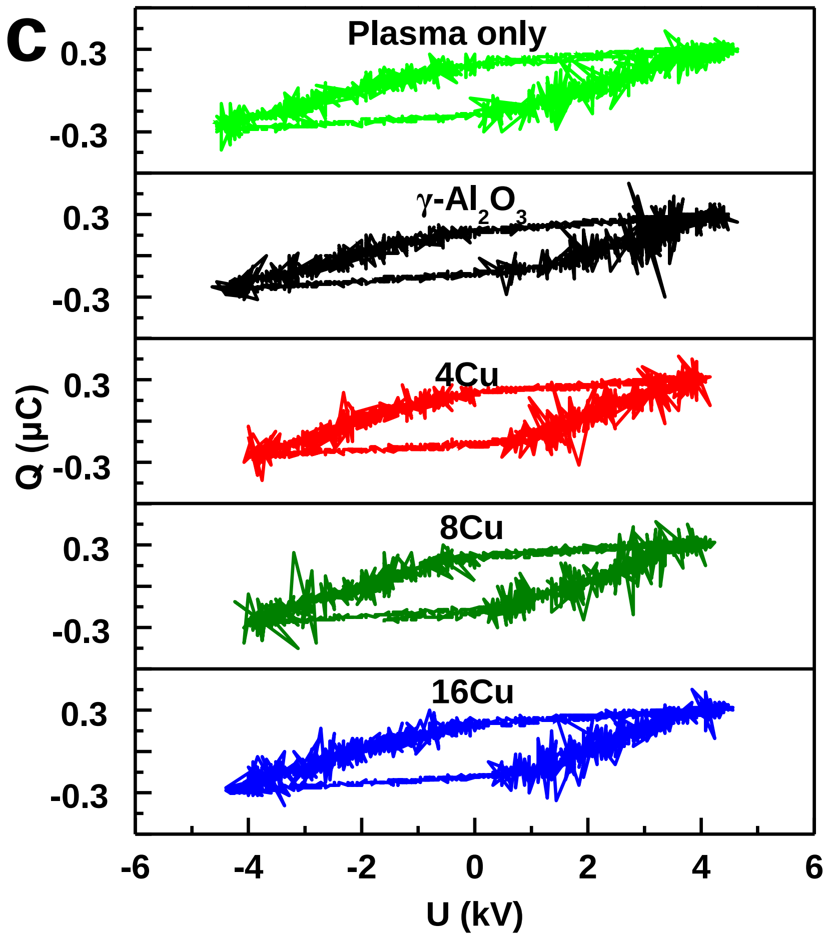


**Figure S4.** Effect of catalysts on the electrical signals of the DBD at 10 W (a) applied voltage, (b) current and (c) Q-U Lissajous figures.

## 3. Catalytic tests of catalysts

### 3.1. Calculation methods of catalytic performance

The conversion of CO ($C_{CO}$) and yield ($Y_{H2}$) of hydrogen were calculated using the following equations, respectively:

$$C_{CO}(\%) = \frac{\text{CO converted (mol s}^{-1}\text{)}}{\text{CO input (mol s}^{-1}\text{)}} \times 100\% \quad (2)$$

$$Y_{H_2}(\%) = \frac{\text{H}_2\text{ produced (mol s}^{-1}\text{)}}{\text{CO input (mol s}^{-1}\text{)}} \times 100\% \quad (3)$$

### 3.2. Calculation methods of kinetics

The turnover frequency (TOF) for the WGS reaction over Cu/γ-$Al_2O_3$ catalysts was calculated at 190 $^{o}$C to ensure low conversion of CO (< 15%). The CO converted ($m$) was determined using equation 4:

$$m\ \text{(mol)} = C_{CO} \times Q \times t \quad (4)$$

where $C_{CO}$ is the CO conversion, $Q$ is the flow rate of CO in mol $s^{-1}$ and $t$ (s) is the time used.

The number of Cu atoms on the catalyst surface ($n$) was calculated using equation 5:

$$n\ \text{(mol)} = \frac{m_{Cu} \times D_{Cu}}{M_{Cu}} \quad (5)$$

where $m_{Cu}$ is the mass of actual Cu loading in the catalyst, $D_{Cu}$ is the dispersion of Cu on the catalyst surface and $M_{Cu}$ is the molar mass of Cu. $D_{Cu}$ is estimated by equation 6:

$$\text{Cu dispersion (\%)} = \frac{\text{Cu}_{surface}\text{(g)}}{\text{Cu}_{actual}\text{(g)}} \times 100 \quad (6)$$

Where $Cu_{actual}$ is the actual Cu loading measured by ICP. While $Cu_{surface}$ is the surface Cu obtained by reactions 7 and 8:[2,3]

$$N_2O + 2Cu_{surface} \rightarrow Cu_2O_{surface} + N_2 \quad (7)$$

$$H_2 + Cu_2O_{surface} \rightarrow 2Cu_{surface} + H_2O \quad (8)$$

Since the amount of $H_2$ consumption can be estimated, the surface Cu is determined considering the stoichiometry of the reaction 8.

The TOF ($s^{-1}$) was calculated using equation 9:

$$\text{TOF}\ (s^{-1}) = \frac{m}{n \times t} \quad (9)$$

The reaction rates ($r$) and rate constants ($k_{Thermal}$ and $k_{Plasma}$) are acquired by:

$$r\ (\text{mol s}^{-1}\ \text{g}^{-1}) = \frac{\text{CO input (mol s}^{-1}) \times C_{CO}}{\text{cat. weight (g)}} \quad (10)$$

$$k_{Thermal} = k_0 \times e^{-\frac{E_a}{RT}} \quad (11)$$

$$k_{plasma} = k_0 \times \exp(-\frac{E_a}{P \times F^{-1}}) \quad (12)$$

Where in the rate constant calculations, Eq. 11 is used for thermal-catalysis, while Eq. 12 is used for plasma-catalysis. $F$ is the total molar flow rate (mol $s^{-1}$), $P$ is the plasma discharge power (W). During the activity tests, the CO conversions were restricted below 15% to decrease the influence of mass transfer and competitive adsorptions between reactants and products.

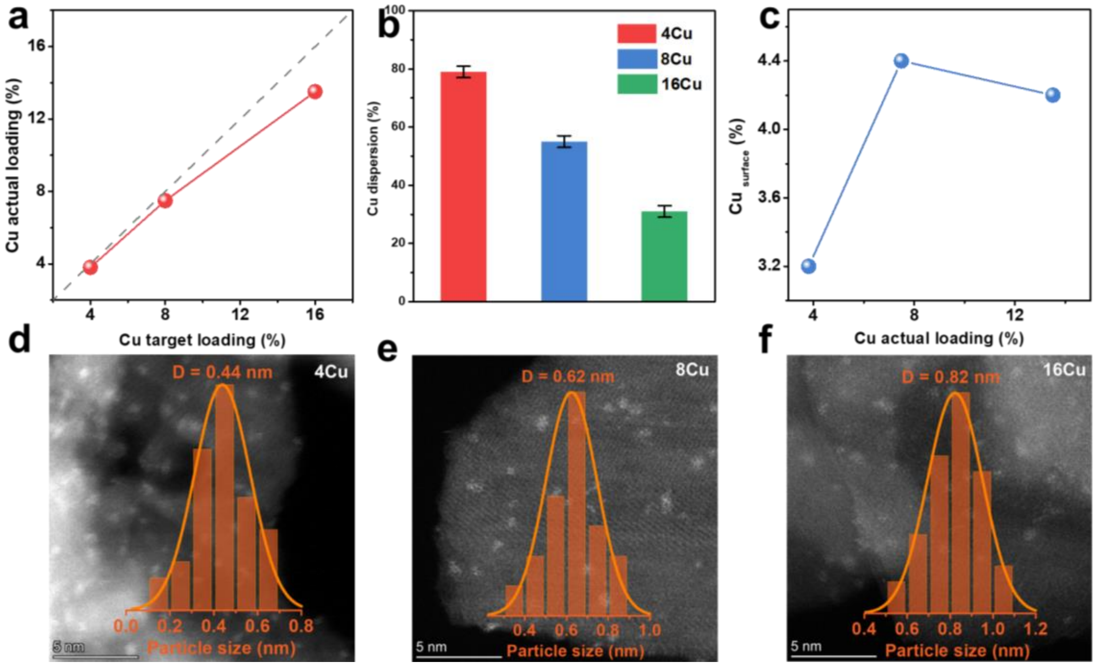

**Figure S5.** (a) Relationship between target and actual (ICP-measured) Cu loading. (b) Cu dispersions of different Cu-based catalysts. (c) surface Cu as a function of actual Cu loading. HAADF-STEM image of (d) 4Cu, (e) 8Cu and (f) 16Cu catalysts after plasma-catalysis.

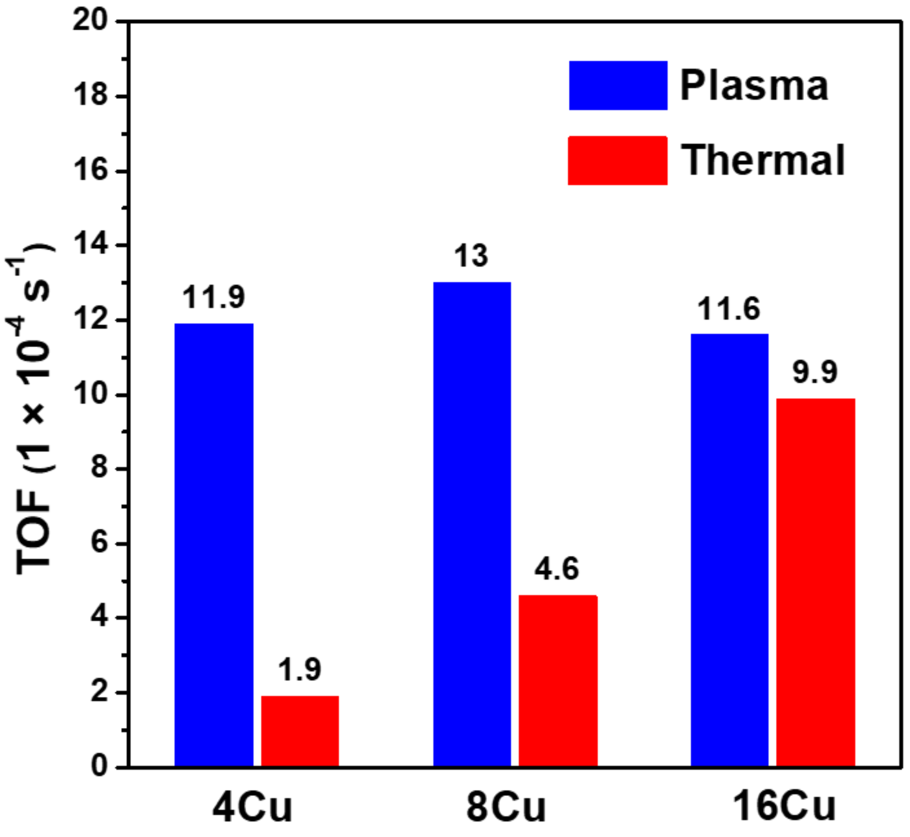


**Figure S6.** Turnover frequency of plasma-catalysis (discharge power: 0.417 W) and thermal-catalysis (temperature: 185 $^{o}C$).

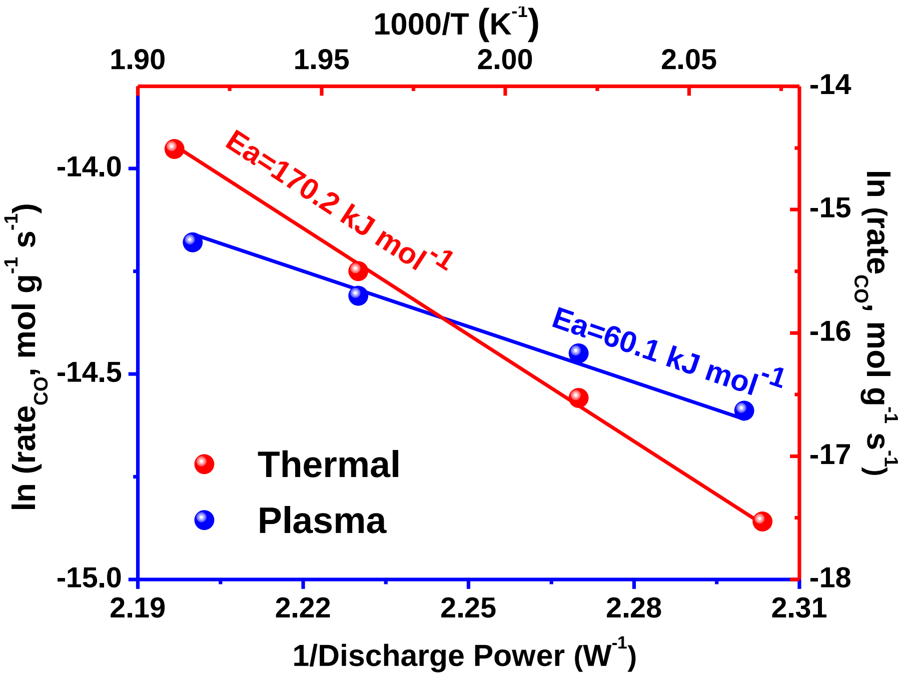


**Figure S7.** Arrhenius plots of Plasma- and thermal-catalytic WGS reaction over γ-$Al_2O_3$ catalysts (CO conversion below 15%).

**Table S1.** The temperatures, discharge powers, CO conversions of TOF and apparent activation energies (Ea) calculations on 4Cu/γ-$Al_2O_3$ catalyst.

| Sample (4Cu) | Temperature (°C) | Discharge power (W) | CO conversion (%) |
|---|---|---|---|
| Thermal activation | 185.7<br>192.1<br>200.9<br>207.8 | - | 0.51<br>0.72<br>1.07<br>1.52 |
| Plasma activation | < 53.2 | 0.417<br>0.422<br>0.429<br>0.435 | 3.21<br>3.55<br>3.89<br>4.30 |

**Table S2.** The temperatures, discharge powers, CO conversions of TOF and apparent activation energies (Ea) calculations on 8Cu/γ-$Al_2O_3$ catalyst.

| Sample (8Cu) | Temperature ($^{o}C$) | Discharge power (W) | CO conversion (%) |
|---|---|---|---|
| Thermal activation | 175.4<br>181.5<br>190.0<br>196.5 | - | 1.02<br>1.46<br>2.07<br>2.97 |
| Plasma activation | < 52.5 | 0.400<br>0.405<br>0.411<br>0.417 | 4.13<br>4.34<br>4.61<br>4.84 |

**Table S3.** The temperatures, discharge powers, CO conversions of TOF and apparent activation energies (Ea) calculations on 16Cu/$\gamma$-$Al_2O_3$ catalyst.

| Sample (16Cu) | Temperature ($^{o}C$) | Discharge power (W) | CO conversion (%) |
|---|---|---|---|
| Thermal activation | 165.6<br>171.4<br>179.5<br>185.7 | - | 1.44<br>1.95<br>2.61<br>3.52 |
| Plasma activation | < 52.7 | 0.408<br>0.413<br>0.420<br>0.426 | 3.70<br>3.97<br>4.21<br>4.52 |

**Table S4.** The temperatures, discharge powers, CO conversions of TOF and apparent activation energies (Ea) calculations on $\gamma$-$Al_2O_3$ catalyst.

| Sample ($\gamma$-$Al_2O_3$) | Temperature ($^{o}C$) | Discharge power (W) | CO conversion (%) |
|---|---|---|---|
| Thermal activation | 210.1<br>222.0<br>237.2<br>250.6 | - | 0.13<br>0.36<br>1.00<br>2.68 |

| | | | |
|---|---|---|---|
| Plasma activation | < 54.8 | 0.435<br>0.441<br>0.448<br>0.455 | 2.48<br>2.85<br>3.28<br>3.73 |

### 3.3. Calculation methods of energy consumption

The energy consumption for $H_2$ production ($EC_{H2}$) and the fuel production efficiency (FPE) were defined as:

$$EC_{H_2}\,(\text{MJ mol}^{-1}) = \frac{\text{Discharge power(kW)}}{1000 \times H_2 \text{ produced (mol s}^{-1})} \quad (13)$$

$$\text{FPE(\%)} = \frac{H_2 \text{ produced (mol s}^{-1}) \times LHV_{H_2} \text{ (kJ mol}^{-1})}{\text{CO converted (mol s}^{-1}) \times LHV_{CO} \text{(kJ mol}^{-1}\text{)+Discharge power(kW)}} \times 100\% \quad (14)$$

Where $LHV_{H2}$ and $LHV_{CO}$ are the low heating value of $H_2$ and CO, respectively.[4]

## 4. *In situ* DRIFTS experiments

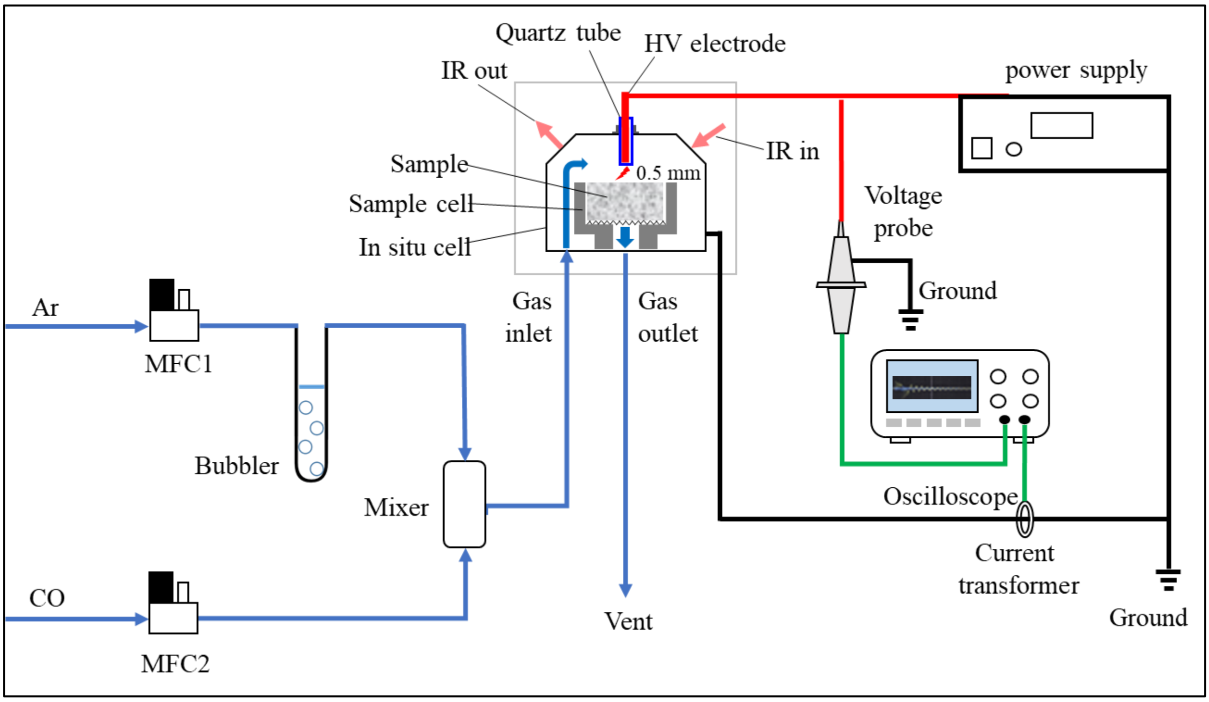


**Figure S8.** Schematic of experimental setup for the DBD-DRIFTS system.

A schematic diagram of the *in situ* plasma-coupled DRIFTS system is shown in Figure S8. The system consisted of a gas supplier unit, an *in situ* DRIFTS reactor, an FTIR spectrometer (Nicolet 50, Thermo Scientific, USA), a high voltage power supply (M10K-08, Suzhou Aikefa, China) and an electrical signal sampling unit. The FTIR spectrometer was equipped with an *in situ* DRIFTS reaction cell (HVC-DRP-5, Harrick, USA) and a narrow-band mercury cadmium telluride (MCT-A) detector with liquid nitrogen cooling for high sensitivity (0.09 $cm^{-1}$) in collecting DRIFT spectra between 4000 and 650 $cm^{-1}$. The *in situ* DRIFTS reaction cell was equipped with a modified observation window and two KBr windows. A one-side sealed quartz tube (inner diameter of 1 mm, outer diameter of 2 mm and the length of 15 mm) was inserted into the observation window. A stainless steel wire (diameter of 0.35 mm) was fixed in the quartz tube by tin, which was served as a high-voltage electrode. And the reaction chamber was served as the ground electrode. The distance between the sample and the tip of sealed quartz tube was 0.5 mm. The discharge waveforms of current and voltage were measured and

recorded by a current transformer (TCP 0030, Tektronix, USA), a voltage probe (P6015A, Tektronix, USA) and a digital phosphor oscilloscope (DPO 3034, Tektronix, USA).

For the CO and $H_2O$ adsorption and reaction experiment with plasma on (discharge frequency, input voltage and output voltage are 500 Hz, 10 V and 6.38 kV, respectivelly) and off, 30 mg powder samples (Cu-based catalysts and γ-$Al_2O_3$) were pretreated for 1 h in Ar at 400 °C (20 mL/min, 99.999%). Then, the gas mixture was cooled to room temperature and stabilized for 10 min, during which the DRIFTS background spectra were collected. Following this, a gas mixture of Ar, CO and steam were injected into the *in situ* reaction cell for 1 h. IR spectra were then collected at 1 min (without discharge) and at different times with or without discharge. The sample temperature (maintained at about 140 $^{o}$C in this study) was elevated through programmed heating using a temperature controller. Thirty-two scans were conducted with a resolution of 4 $cm^{-1}$, and the DRIFT spectra were analyzed using OMNIC software. The Kubelka-Munk function was used to convert the obtained spectra into absorption spectra, with intensities linearly related to the amount of adsorption.

## 5. DFT calculations

### 5.1. Calculation details

All the DFT calculations in this work were carried out using the Vienna ab-initio simulation package (VASP) with a projector augmented wave (PAW) method. The nonlocal exchange correlation energy was described using the generalized gradient approximation (GGA) and Perdew-Burke-Ernzerhof (PBE) functional. A plane wave basis set with a 400-eV cut-off kinetic energy was used and a (2 × 2 × 1) Monkhorst-Pack k-point grid was employed to sample the surface Brillouin zone. Both the cutoff energy and the k-point grid were performed to ensure converged results. Very strict convergence criteria ($10^{-5}$ eV for the total energy change and $10^{-3}$ eV/Å for the forces) were used. A slab model consisting of the γ-$Al_2O_3$ (110) surface (monoclinic non-spinel model) and copper atoms was used to simulate the Cu/γ-$Al_2O_3$ catalyst.[5-8] As shown in Figure S9, the γ-$Al_2O_3$ surface consisted of 4 atomic layers with a (1 × 1) periodic supercell. A vacuum region of 10Å in the z direction was included to prevent unintended interactions of adsorbed species with the bottom layer of the slab in the subsequent replica. During the optimization processes, the bottom two layers were constrained in their bulk positions, while the remaining two layers, along with the adsorbed species, were allowed to relax. We built three models containing varying amounts of Cu atoms (1, 2, 4) on the γ-$Al_2O_3$ surface to represent potential sites of γ-$Al_2O_3$ supported Cu catalysts, as shown in Figure S9. As a single Cu atom can bind to several different surface sites, the stabilities of different Cu doped sites ($Cu_1$-$O_{2C12}$, $Cu_1$-$O_{2C23}$, $Cu_1$-$Al_{3C}O_{3C1}$) were compared using the total energies of the possible structures that could be formed. The results show that $Cu_1$-$O_{2C23}$ (a single Cu atom bonded with $O_{2C-2}$ and $O_{2C-3}$) exhibited the most stable $Cu_1$ structure. Subsequently, the structures of $Cu_2$/γ-$Al_2O_3$ and $Cu_4$/γ-$Al_2O_3$ were built based on relevant literature.[7,8]

The climbing-image nudged elastic band (CI-NEB) method was used to search for the transition states of every elementary reaction in the low-temperature WGS, with convergence

criteria set at a force of 0.05 eV/Å on each atom. All obtained transition states were verified through vibrational frequency calculations, confirming the presence of only one imaginary mode during each run, indicating the correct transition configuration had been achieved. Zero-point energies and entropy corrections were incorporated in all DFT calculations at 423 K. In the OH-participated routes, the energies were corrected using the equation $G = EH + E_{ZPE} - TS + RTln(P^{OH}/P^0)$, where the partial pressure of the OH species $P^{OH}$ was set at $0.1P^0$ ($P^0$ represents atmosphere pressure, T is 423 K). Two typical WGS reaction mechanisms, namely the redox and carboxyl mechanisms, were introduced to investigate the reaction mechanisms under plasma-catalytic conditions. Hydroxyl molecules were added in specific elementary reaction studies to simulate species with high energy generated by the plasma, including $e^-$, OH species and $H_2O^+$.

The banding energies of copper atoms and the adsorption energies of adsorbates were defined as follows:

$$E_{bin} = E_{\mathrm{Cu}_{x(x=1,2,4)}/\gamma\text{-}\mathrm{Al_2O_3}} - E_{\mathrm{Cu}_x} - E_{\gamma\text{-}\mathrm{Al_2O_3}} \tag{15}$$

Where $E_{Cux/\gamma\text{-}Al2O3}$ represents the total energy of copper atoms with the $\gamma$-$Al_2O_3$ surface, while $E_{Cux}$ and $E_{\gamma\text{-}Al2O3}$ are the total energies of free $Cu_x$ and the clean $\gamma$-$Al_2O_3$ slab, respectively.

$$E_{ads} = E_{ad/\gamma\text{-}\mathrm{Al_2O_3}} - E_{ad} - E_{\gamma\text{-}\mathrm{Al_2O_3}} \tag{16}$$

Where $E_{ad/\gamma\text{-}Al2O3}$ is the energy of the optimized adsorption system of adsorbates and the $\gamma$-$Al_2O_3$ support, $E_{ad}$ is the energy of adsorbed species in the gas phase.

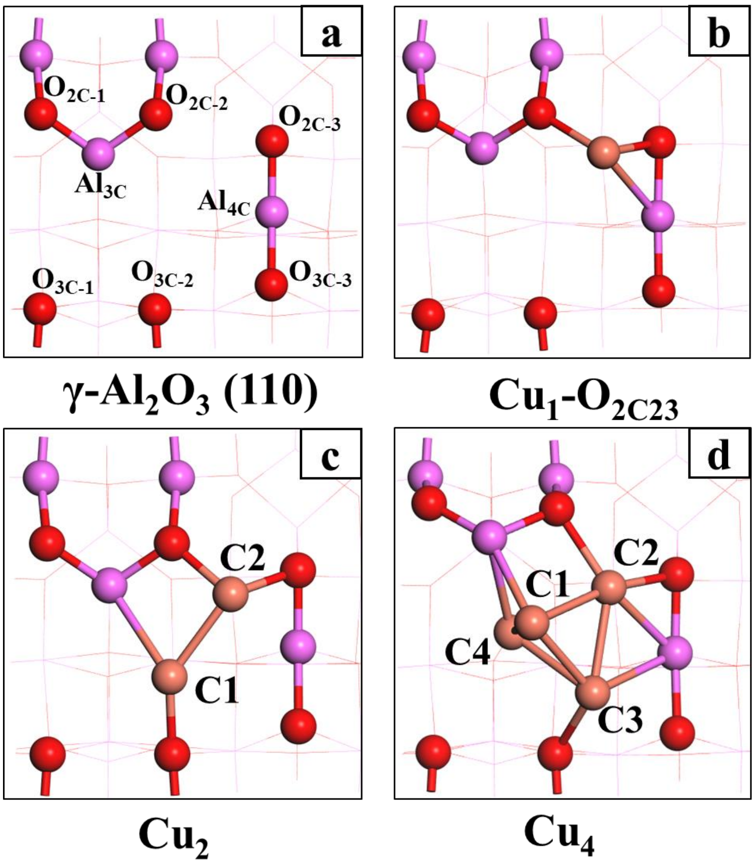


**Figure S9.** The established models of (a) γ-$Al_2O_3$ (110) surface, (b) $Cu_1$/γ-$Al_2O_3$, (c) $Cu_2$/γ-$Al_2O_3$ and (d) $Cu_4$/γ-$Al_2O_3$ catalyst surface sites. C1, C2, C3 and C4 in pictures (c) and (d) denote different Cu adsorption sites. Pink, red and orange balls represent Al, O and Cu atoms, respectively.

## 5.2. Calculation results

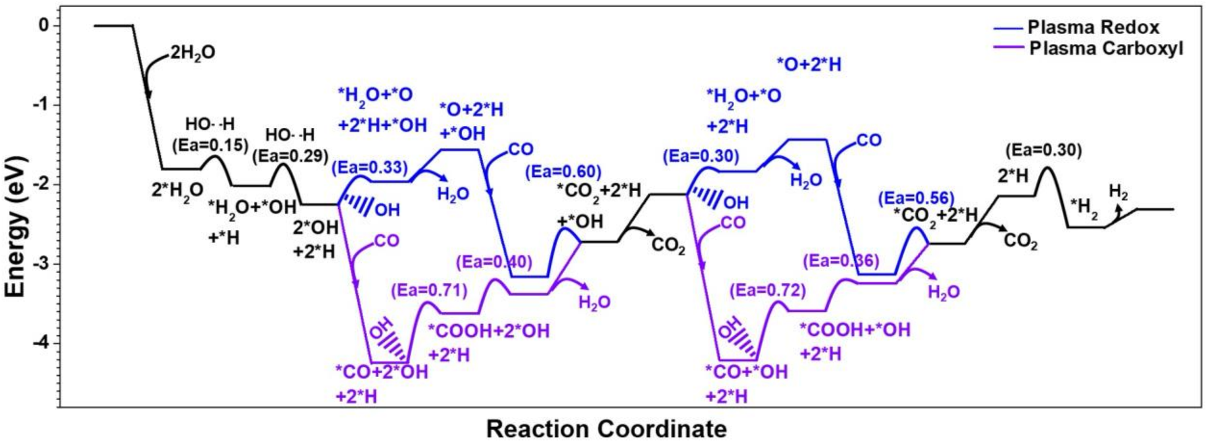

**Figure S10.** Plasma reaction routes and energy profiles of the WGS reaction on the $Cu_2/\gamma$-$Al_2O_3$ model.

**Table S5.** Adsorption energies of OH, $H_2O$ and CO on different sites of $Cu_1/\gamma$-$Al_2O_3$, $Cu_2/\gamma$-$Al_2O_3$ and $Cu_4/\gamma$-$Al_2O_3$

| Reactants | $Cu_1$ (eV) | | $Cu_2$ (eV) | | | $Cu_4$ (eV) | | | | |
|---|---|---|---|---|---|---|---|---|---|---|
| and sites | Cu | $Al_{3C}$ | C1 | C2 | $Al_{3C}$ | C1 | C2 | C3 | C4 | $Al_{3C}$ |
| OH | -0.88 | -1.40 | -0.98 | -0.88 | -1.54 | -0.61 | -0.88 | -0.88 | -0.88 | -1.13 |
| $H_2O$ | -0.35 | -1.75 | - | -0.40 | -1.43 | - | -0.52 | -0.46 | - | -1.23 |
| CO | -1.52 | -0.64 | -1.45 | -1.51 | -0.62 | -0.80 | -1.27 | -1.10 | -0.92 | -0.50 |

**Table S6.** Redox and Carboxyl mechanisms

| Redox | Carboxyl |
|---|---|
| $H_2O + CO + 2^* \rightarrow {}^*H_2O + {}^*CO$ | $H_2O + CO + 2^* \rightarrow {}^*H_2O + {}^*CO$ |
| $^*H_2O + {}^* \rightarrow {}^*OH + {}^*H$ | $^*H_2O + {}^* \rightarrow {}^*OH + {}^*H$ |
| $^*OH\ ({}^*H_2O)^a + {}^* \rightarrow {}^*O + {}^*H$ | $^*CO + {}^*OH\ ({}^*H_2O)^a \rightarrow {}^*COOH + {}^*$ |
| $^*OH\ ({}^*H_2O)^a + {}^*OH\ ({}^*H_2O)^a \rightarrow {}^*H_2O + {}^*O$ | $^*CO + {}^*OH\ ({}^*H_2O)^b \rightarrow {}^*COOH + {}^*$ |
| $OH(H_2O)^b + {}^*OH\ ({}^*H_2O)^a \rightarrow {}^*H_2O + {}^*O$ | $^*COOH + {}^* \rightarrow {}^*CO_2 + {}^*H$ |
| $^*CO + {}^*O \rightarrow {}^*CO_2 + {}^*$ | $^*COOH + {}^*OH \rightarrow {}^*CO_2 + {}^*H_2O$ |
| $^*H + {}^*H \rightarrow {}^*H_2 + {}^*$ | $^*H + {}^*H \rightarrow {}^*H_2 + {}^*$ |

Reactions involved OH species were generated by different mechanisms, $^aH_2O$ represents dissociation on the catalyst surface and $^bH_2O$ represents dissociation directly in the plasma condition. * denotes a vacant site and *X represents the adsorbed species.

**Table S7.** Calculated reaction energies (ΔE) and energy barriers ($E_a$) on $Cu_1/\gamma$-$Al_2O_3$, $Cu_2/\gamma$-$Al_2O_3$ and $Cu_4/\gamma$-$Al_2O_3$ surfaces via thermal routes.

| Elementary reactions | $Cu_1$ | $Cu_2$ | $Cu_4$ |
|---|---|---|---|

| | | ΔE (eV) | $E_a$ (eV) | ΔE (eV) | $E_a$ (eV) | ΔE (eV) | $E_a$ (eV) |
|---|---|---|---|---|---|---|---|
| $2H_2O + 2^* \rightarrow 2^*H_2O$ | | -2.20 | - | -1.80 | - | -1.65 | - |
| $^*H_2O + ^* \rightarrow ^*OH + ^*H$ | | -0.3 | 0.18 | -0.2 | 0.15 | -0.1 | 0.24 |
| $^*H_2O + ^* \rightarrow ^*OH + ^*H$ | | -0.33 | 0.39 | -0.25 | 0.29 | -0.42 | 0.45 |
| T-R | $^*OH + ^*OH \rightarrow ^*H_2O + ^*O$ | 0.47 | 0.86 | 0.29 | 0.75 | 0.52 | 0.93 |
| | $^*H_2O \rightarrow H_2O+^*$ | 0.40 | - | 0.40 | - | 0.40 | - |
| | $CO + ^* \rightarrow ^*CO$ | -1.52 | - | -1.60 | - | -1.58 | - |
| | $^*CO + ^*O \rightarrow ^*CO_2 + ^*$ | 0.54 | 0.82 | 0.39 | 0.56 | 0.33 | 0.71 |
| T-C | $CO + ^* \rightarrow ^*CO$ | -2.2 | - | -2.09 | - | -1.94 | - |
| | $^*CO + ^*OH \rightarrow ^*COOH + ^*$ | 0.52 | 1.21 | 0.62 | 1.02 | 0.63 | 1.11 |
| | $^*COOH + ^*OH \rightarrow ^*CO_2 + ^*H_2O$ | 0.35 | 0.51 | 0.25 | 0.40 | 0.44 | 0.35 |
| | $^*H_2O \rightarrow H_2O+^*$ | 0.40 | - | 0.40 | - | 0.40 | - |
| $^*CO_2 \rightarrow CO_2 + ^*$ | | 0.60 | - | 0.60 | - | 0.60 | - |
| $^*H + ^*H \rightarrow ^*H_2 + ^*$ | | -0.51 | 0.44 | -0.40 | 0.30 | -0.38 | -0.33 |
| $^*H_2 \rightarrow H_2 + ^*$ | | 0.20 | - | 0.20 | - | 0.20 | - |

T-R and T-C represent the thermal redox route and thermal carboxyl route, respectively. * denotes a vacant site and *X represents the adsorbed species.

**Table S8.** Calculated reaction energies (ΔE) and energy barriers ($E_a$) on $Cu_1$/γ-$Al_2O_3$, $Cu_2$/γ-$Al_2O_3$ and $Cu_4$/γ-$Al_2O_3$ surfaces via plasma routes.

| Elementary reactions | | $Cu_1$ | | $Cu_2$ | | $Cu_4$ | |
|---|---|---|---|---|---|---|---|
| | | ΔE (eV) | $E_a$ (eV) | ΔE (eV) | $E_a$ (eV) | ΔE (eV) | $E_a$ (eV) |
| $2H_2O + 2^* \rightarrow 2^*H_2O$ | | -2.20 | - | -1.80 | - | -1.65 | - |
| $^*H_2O + ^* \rightarrow ^*OH + ^*H$ | | -0.3 | 0.18 | -0.2 | 0.15 | -0.1 | 0.24 |
| $^*H_2O + ^* \rightarrow ^*OH + ^*H$ | | -0.33 | 0.39 | -0.25 | 0.29 | -0.42 | 0.45 |
| P-R | $^*OH + ^*OH$ [(a)] $\rightarrow ^*H_2O + ^*O$ | 0.47 | 0.56 | 0.29 | 0.33 | 0.52 | 0.43 |
| | $^*H_2O \rightarrow H_2O+^*$ | 0.40 | - | 0.40 | - | 0.40 | - |
| | $CO + ^* \rightarrow ^*CO$ | -1.52 | - | -1.61 | - | -1.58 | - |
| | $^*CO + ^*O \rightarrow ^*CO_2 + ^*$ | 0.54 | 0.82 | 0.39 | 0.60 | 0.33 | 0.71 |

| | | | | | | | |
|---|---|---|---|---|---|---|---|
| P-C | $CO + * \rightarrow *CO$ | -2.2 | - | -2.09 | - | -1.94 | - |
| | $*CO + *OH$ [(a)] $\rightarrow *COOH + *$ | 0.52 | 0.83 | 0.62 | 0.71 | 0.63 | 0.92 |
| | $*COOH + *OH \rightarrow *CO_2 + *H_2O$ | 0.35 | 0.51 | 0.25 | 0.40 | 0.44 | 0.35 |
| | $*H_2O \rightarrow H_2O+*$ | 0.40 | - | 0.40 | - | 0.40 | - |
| $*CO_2 \rightarrow CO_2 + *$ | | 0.60 | - | 0.60 | - | 0.60 | - |
| $*H + *H \rightarrow *H_2 + *$ | | -0.51 | 0.44 | -0.40 | 0.30 | -0.38 | -0.33 |
| $*H_2 \rightarrow H_2 + *$ | | 0.20 | - | 0.20 | - | 0.20 | - |

P-R and P-C represent the plasma redox route and plasma carboxyl route, respectively. *X represents the adsorbed species.

## References


(1) Wang, Y.; Craven, M.; Yu, X.; Ding, J.; Bryant, P.; Huang, J.; Tu, X. Plasma-Enhanced Catalytic Synthesis of Ammonia over a Ni/$Al_2O_3$ Catalyst at Near-Room Temperature: Insights into the Importance of the Catalyst Surface on the Reaction Mechanism. *ACS Catal.* **2019**, *9*, 10780-10793.

(2) López-Suárez, F. E.; Bueno-López, A.; Illán-Gómez, M. J., Cu/$Al_2O_3$ catalysts for soot oxidation: Copper loading effect. *Appl. Catal. B Environ*. **2008**, *84*, 651-658.

(3) Gervasini, A.; Bennici, S., Dispersion and surface states of copper catalysts by temperature-programmed-reduction of oxidized surfaces (s-TPR). *Appl. Catal. A Gen*. **2005**, *281*, 199-205.

(4) Mei, D.; Ashford, B.; He, Y.; Tu, X. Plasma-catalytic reforming of biogas over supported Ni catalysts in a dielectric barrier discharge reactor: Effect of catalyst supports. *Plasma Process. Polym.* **2017**, *14*, 1600076

(5) Song, Z.; Wang, B.; Yu, J.; Ma, C.; Qu, Q.; Zeng, Z.; Xiang, J.; Hu, S.; Sun, L. Removal of $Hg^0$, NO, and $SO_2$ by the surface dielectric barrier discharge coupled with Mn/Ce/Ti-based catalyst. *Appl. Surf. Sci.* **2016**, *387*, 341-350.

(6) Liu, C.; Yang, B.; Tyo, E.; Seifert, S.; DeBartolo, J.; von Issendorff, B.; Zapol, P.; Vajda, S.; Curtiss, L. A. Carbon Dioxide Conversion to Methanol over Size-Selected $Cu_4$ Clusters at Low Pressures. *J. Am. Chem. Soc.* **2015**, *137*, 8676-8679.

(7) Pan, Y. X.; Liu, C. J.; Wiltowski, T. S.; Ge, Q. $CO_2$ adsorption and activation over γ-$Al_2O_3$-supported transition metal dimers: A density functional study. *Catal. Today*. **2009**, *147*, 68-76.

(8) Digne, M. Use of DFT to achieve a rational understanding of acid–basic properties of γ-alumina surfaces. *J. Catal*. **2004**, *226*, 54-68.
